# Prebiotic Effect of Fructooligosaccharides from *Morinda officinalis* on Alzheimer’s Disease in Rodent Models by Targeting the Microbiota-Gut-Brain Axis

**DOI:** 10.3389/fnagi.2017.00403

**Published:** 2017-12-08

**Authors:** Diling Chen, Xin Yang, Jian Yang, Guoxiao Lai, Tianqiao Yong, Xiaocui Tang, Ou Shuai, Gailian Zhou, Yizhen Xie, Qingping Wu

**Affiliations:** ^1^State Key Laboratory of Applied Microbiology Southern China, Guangdong Provincial Key Laboratory of Microbial Culture Collection and Application, Guangdong Open Laboratory of Applied Microbiology, Guangdong Institute of Microbiology, Chinese Academy of Sciences, Guangzhou, China; ^2^Department of Pharmacy, The Fifth Affiliated Hospital of Guangzhou Medical University, Guangzhou, China; ^3^Guangxi University of Chinese Medicine, Nanning, China; ^4^Guangdong Yuewei Edible Fungi Technology Co., Ltd., Guangzhou, China

**Keywords:** fructooligosaccharides, prebiotics, Alzheimer’s disease, behavior, microbiota-gut-brain axis

## Abstract

Gut microbiota influences the central nervous system disorders such as Alzheimer’s disease (AD). The prebiotics and probiotics can improve the host cognition. A previous study demonstrated that fructooligosaccharides from *Morinda officinalis* (OMO) exert effective memory improvements in AD-like animals, thereby considered as potential prebiotics; however, the underlying mechanism still remains enigma. Thus, the present study investigated whether OMO is effective in alleviating AD by targeting the microbiota-gut-brain axis. OMO was administered in rats with AD-like symptoms (D-galactose- and Aβ_1-42_-induced deficient rats). Significant and systematic deterioration in AD-like animals were identified, including learning and memory abilities, histological changes, production of cytokines, and microbial community shifts. Behavioral experiments demonstrated that OMO administration can ameliorate the learning and memory abilities in both AD-like animals significantly. AD parameters showed that OMO administration cannot only improve oxidative stress and inflammation disorder, but also regulate the synthesis and secretion of neurotransmitter. Histological changes indicated that OMO administration ameliorates the swelling of brain tissues, neuronal apoptosis, and down-regulation of the expression of AD intracellular markers (Tau and Aβ_1-42_). 16S rRNA sequencing of gut microbiota indicated that OMO administration maintains the diversity and stability of the microbial community. In addition, OMO regulated the composition and metabolism of gut microbiota in inflammatory bowel disease (IBD) mice model treated by overdosed antibiotics and thus showed the prebiotic potential. Moreover, gut microbiota plays a major role in neurodevelopment, leading to alterations in gene expression in critical brain and intestinal regions, thereby resulting in perturbation to the programming of normal cognitive behaviors. Taken together, our findings suggest that the therapeutic effect of the traditional medicine, *M. officinalis*, on various neurological diseases such as AD, is at least partially contributed by its naturally occurring chemical constituent, OMO, via modulating the interaction between gut ecology and brain physiology.

## Introduction

Gut microbiota is associated with several diseases, including neurodegenerative diseases such as Parkinson’s disease (PD) and Alzheimer’s disease (AD) (Petra et al., 2015; Jiang et al., 2017). Notably, the microbiota-gut-brain axis is a bi-directional communication system that is not fully understood; however, it is known to include neural, immune, endocrine, and metabolic pathways. Studies in germ-free animals and those exposed to pathogenic microbial infections, antibiotics, probiotics, or fecal microbiota transplantation suggest the link of gut microbiota with host cognition or AD-related pathogenesis (Pistollato et al., 2016; Harach et al., 2017; Liu et al., 2017; Rieder et al., 2017; Russo et al., 2017).

Several studies have supported the theory of the occurrence of a pathway of communication between the gut and the brain, modulated by gut microbiota (Gareau, 2014; Mayer et al., 2014). It has been speculated that targeting the microbiota can affect the behavior and modulate brain plasticity and cognitive functions while aging (Leung and Thuret, 2015). Some studies demonstrated that gut-targeted intervention by consuming *lactic acid bacteria* such as those in yogurt, could improve or delay the onset of cognitive decline associated with aging (Jung et al., 2012; Choi et al., 2015; Scott et al., 2017). Therefore, using probiotics for ameliorating the cognitive and behavioral disorders could be a potential treatment.

The hypothesis that the microbiota-gut-brain axis (Rhee et al., 2009) plays a critical role in health and disease, including neuropsychiatric disorders (Liu et al., 2017), is rapidly progressing. Nurturing a beneficial gut microbiome with prebiotics, such as fructooligosaccharides and inulin, is an appealing but under-investigated microbiota-induced manipulation. A previous study showed that the prebiotic treatment could modify the behavior across domains relevant to anxiety, depression, cognition, stress response, and social behavior (Burokas et al., 2017). Previous findings strengthened the evidence supporting the therapeutic targeting of gut microbiota in brain-gut axis disorders, thereby opening new prospects in the field of nutritional neuropsychopharmacology. Thus, it is imperative to develop novel and effective drugs or foods with prebiotic effects from natural resources.

*Morinda officinalis* How. (*M. officinalis*), as a Chinese traditional natural herbal medicine, contains a number of active components. Reportedly, the content of saccharides in *M. officinalis* radix is 49.79–58.25%, which is highly composed of oligosaccharides, such as inulin-type hexasaccharide exerting antidepressant effects in the model systems (Cai et al., 1996; Li et al., 2001). This phenomenon can be effectuated by up-regulating the expression of neurotrophic factors and/or down-regulating the [Ca^2+^]_i_ overloading (Li et al., 2004). Bajijiasu, another oligosaccharide, protects PC12 cells from Aβ_25-35_-induced neurotoxicity (Chen et al., 2013), ameliorates the cognitive deficits induced by D-galactose in mice, and protects against ischemia-induced neuronal damage or death (Tan et al., 2000a,b). Our previous study suggested that oligosaccharide extracted from *M. officinalis* (OMO) might inhibit the oxidative stress and neuronal apoptosis, restore normal energy metabolism, as well as, increase the cell viability and mitochondrial membrane potential in AD animal models significantly (Chen et al., 2014a). However, the underlying mechanism is yet to be elucidated.

Alzheimer’s disease is the most common neurodegenerative disorder, affecting approximately >5% of the worldwide population aged >65 years, annually. AD is a chronic neurodegenerative disease that frequently exhibits a slow progression accompanied by a greater recession of the disease over a period. Although the cause of AD is poorly understood, among various biochemical and morphological events, the presence of neurofibrillary tangles, senile plaques, and neuronal and synaptic loss are considerably noted (Chen et al., 2017). Several pieces of evidence have confirmed that the accumulation of intracellular β-amyloid (Aβ) may be an early event in the development of AD. Aβ is a peptide comprised of 36–43 amino acids formed by a large transmembrane glycoprotein, such as amyloid precursor protein (APP), expressed on the cell. Aβ may activate the inflammatory and neurotoxic processes, including the excessive generation of free radicals and oxidative damage among intracellular proteins and other macromolecules (Abbott, 2011). D-galactose can form advanced glycation end products (AGEs). The administration of D-galactose to human populations can induce cognitive deficits and disruptions in the synaptic communication. Thus, D-galactose-treated rats with synaptic disruption and memory impairment have been extensively used as model rodents (Zhan et al., 2014; Gao et al., 2016; Huang et al., 2016; Li et al., 2016). In our previous, we have used these two models to screen the effective compounds for curing the AD (Chen et al., 2014b, 2017). Thus, in this study, the two AD-like models were induced by D-galactose and Aβ, respectively.

Herein, we investigated whether OMO is effective in alleviating AD by targeting the microbiota-gut-brain axis. OMO was administered in rats exhibiting AD-like symptoms induced by D-galactose and Aβ_1-42_, respectively. Some indexes and histological deterioration, including learning and memory abilities, histological alterations, production of cytokines, microbial communities, and transcriptome in small intestine and brain, were identified in AD-like animals. And the prebiotics were directly evaluated in an overdose antibiotics-treated trinitro-benzene-sulfonic acid (TNBS)-induced mice model.

## Materials and Methods

### Animal Models and Treatments

Adult male Sprague–Dawley rats (180–220 g) and C57 mice (18–22 g, 10-moth-old) obtained from the Center of Laboratory Animal of Guangdong Province, SCXK [Yue] 2008-0020, SYXK [Yue] 2008-0085) were pair-housed in plastic cages in a temperature-controlled (25°C) colony room at a 12/12 h light/dark cycle. Food and water were available *ad libitum*. All experimental protocols were approved by the Center of Laboratory Animals of the Guangdong Institute of Microbiology (GT-IACUC20160426). All efforts were made to minimize the number of animals used.

#### D-Galactose-Induced Deficient Rats and Treatment

The rats were randomly divided into four groups as follows: control group received distilled water orally, model group received intraperitoneal injection (i.p.) of 100 mg/kg/d D-galactose (Zhong et al., 2016; Chen et al., 2017; Liang et al., 2017), low-dose group was administered D-galactose (100 mg/kg/d) by i.p. and gavage at a dosage of 50 mg/[kg⋅d] OMO, and high-dose group received D-galactose (100 mg/kg/d) by i.p. and gavage at a dosage of 100 mg/[kg⋅d] OMO daily in the morning. Every group consisted of eight animals, and the duration of the procedure was 8 weeks.

#### Aβ_1-42_-Induced Deficient Rats and Treatment

The procedures were similar to those described previously (Chen et al., 2014b). Rats were anesthetized using 30 g/L pentobarbital sodium (40 mg/kg, i.p.; Sigma–Aldrich) and placed in a stereotaxic frame (RWD Life Science Co., Ltd., Shenzhen, China). The hair was shaved, scalp opened, and holes drilled with an electric dental drill (brushless motor, 30,000 rpm) according to the mouse brain atlas (AP-3.6 mm, ML ± 2.5 mm, DV3.0 mm). Then, 5 μL (10 μg) Aβ_1-42_ in a fibrillar state (Chen et al., 2013) was slowly injected into the CA1 region of the hippocampus over a 5-min period in one hole, and the needle was retained inside for an additional 5 min. Subsequently, the wound was sutured, and penicillin (30 U/kg) was injected intramuscularly to protect against infection. Finally, the rats were isolated in a warm box until consciousness was recovered.

After 15 days, the rats were screened with water maze tests to identify the animals that were appropriate models, followed by random categorization into four groups as follows: control group (received saline and distilled water orally), model group (received Aβ_1-42_ and distilled water orally), low-dose group (received Aβ_1-42_ and OMO 50 mg/[kg⋅d] orally), high-dose group (received Aβ_1-42_ and OMO 100 mg/[kg⋅d] orally). Every group consisted of seven animals, and the duration of the experiments was 28 days.

### Water Maze Tests

The spatial learning and memory abilities of the rats were tested using the Morris water maze (MWM, DMS-2, Chinese Academy of Medical Sciences Institute of Medicine). The MWM consisted of a circular opaque fiberglass pool (200 cm diameter) filled with water (25 ± 1°C). The pool was surrounded by light blue curtains, and three distal visual cues were fixed on the curtains. A total of four floor light sources of equal power provided uniform illumination to the pool and testing room. A CCD camera was placed above the center of the pool in order to record the swim paths of the animals. The video output was digitized by an EthoVision tracking system (Noldus, Leesburg, VA, United States). The tests included three periods: initial spatial training, spatial reversal training, and the probe test; the procedures were same as those described previously (Chen et al., 2014b).

### Evaluation of AD Parameters

The appearance, behavior, and fur color of the animals were observed and documented daily. The weights of the animals were measured every 3 days during the period of drug administration. Following the MWM, the blood and serum were acquired, and the brains of the animals were dissected. Routine index and cytokines (Wang et al., 2016), including the production of cytokines interleukins [(1L)-1α, 1L-2, 1L-8, 1L-10, 1L-11, IL-12], tumor necrosis factor (TNF)-γ, TNF-α, vascular endothelial growth factor (VGEF), human macrophage inflammatory protein-1α (MIP-α), and macrophage colony-stimulating factor (M-CSF), activities of malondialdehyde (MDA), total superoxide dismutase (T-SOD), catalase (CAT), glutathione reductase (GSH-Px), and levels of acetylcholine (ACh), acetylcholinesterase (AChE), and Na^+^/K^+^-ATPase, and some monoamine neurotransmitters, were measured.

A total of three brains, small intestine, and other tissues from each group were fixed in 4% paraformaldehyde and prepared as paraffin sections that were stained with hematoxylin-eosin (H&E) and immunohistochemistry (IHC) before examining under light microscopy (Zeng et al., 2013; Chen et al., 2014b).

### Evaluation of Prebiotic Effects of OMO in TNBS-Induced Mice

After 24 h fasting, the mice were anesthetized by intraperitoneal injection of 2% sodium pentobarbital (0.2 ml/100 g), followed by intubation (latex tubing of 2 mm diameter, lubricated with edible oil before usage) from the anus, gently inserted into the lumen about 4.0 cm. Subsequently, 150 mg/kg of TNBS (Sigma–Aldrich, St. Louis, MO, United States, solubilized in 50% ethanol) solution was injected through the latex tubing, the rats were hanged upside down for 30 s to ensure complete seepage of the mixture into the lumen without leakage (Wei et al., 2017). Then, the animals were randomly divided into nine groups (*n* = 9): control, model, model and high-dose antibiotics, OMO (100 mg/kg/d), *Bifidobacterium*, OMO and high-dose antibiotics, OMO and *Bifidobacterium*, *Bifidobacterium* and high-dose antibiotics, OMO and *Bifidobacterium* and high-dose antibiotics. All the antibiotics were administered for 4 days, then inflammatory bowel disease (IBD) was induced with TNBS, followed 7-day drug treatments and TNBS induction, followed by an additional 4-day drug treatments.

Consequently, the mice were anesthetized by intraperitoneal injection of 2% sodium pentobarbital (0.25 ml/100 g). The blood plasma was collected by abdominal aortic method and serum by centrifugation (1500 r/min, 10 min). Then, the serum was assessed for the production of cytokines GM-CSF (granulocyte-macrophage colony-stimulating factor), TNF-γ, 1L-10, IL-12, 1L-17α, 1L-4, TNF-α, and VGEF. The colon and spleen obtained from the rats were fixed in 4% paraformaldehyde at pH 7.4 for further pathological observations, and the cecum contents were collected for 16S rRNA gene-based analysis.

### Microbiome Analysis

Fresh fecal samples were collected before fasting of the rats and stored at -80°C. Frozen microbial DNA was isolated from mice cecal sample with total mass ranging from 1.2 to 20.0 ng and preserved at -20°C. The microbial 16S rRNA genes were amplified using the forward primer 5′-CCTAYGGGRBGCASCAG-3′ and reverse primer 5′-GGACTACNNGGGTATCTAAT-3′ for rats. Each amplified product was concentrated via solid-phase reversible immobilization and quantified by electrophoresis using an Agilent 2100 Bioanalyzer (Agilent, United States). After quantification of DNA concentration by NanoDrop, each sample was diluted to 1 × 10^9^ molecules/μL in TE buffer and pooled. Subsequently, 20 μL of the pooled mixture was used for sequencing on Illumina MiSeq sequencing platform according to the manufacturer’s instructions. The resulting reads were analyzed as described previously (Ling et al., 2014).

### Transcriptome Analysis

The RNA-seq transcriptome library was prepared using the TruSeq^TM^ RNA Sample Preparation Kit (Illumina, San Diego, CA, United States). *De novo* assembly and annotation identified the differentially expressed genes (DEGs) between different treatments; the expression level of each transcript was measured according to the fragments/kb of exon per million mapped reads method. RSEM^1^ was used to quantify the abundance of genes and isoforms. The R statistical package software EdgeR^2^ was used for the analysis of differential expression. Functional enrichment analysis was performed to identify the DEGs enriched significantly in Gene Ontology (GO) and metabolic pathways at Bonferroni-corrected *p*-value ≤ 0.05 as compared to the whole- transcriptome background. GO functional enrichment and KEGG pathway analyses were performed using Goatools^3^ and KOBAS^4^, respectively (Wang et al., 2010; Cabili et al., 2011; Sun et al., 2013; Trapnell et al., 2013).

### Statistical Analysis

All data are described as the means ± standard deviations (SD) of at least three independent experiments. The significant differences between treatments were analyzed by one-way analysis of variance (ANOVA) test at *p* < 0.05 using statistical package for the social sciences (SPSS, Abacus Concepts, Berkeley, CA, United States) and Prism5 (GraphPad, San Diego, CA, United States) software.

## Results

### Effects of OMO in D-Galactose-Induced Deficient Rats

#### Antioxidative and Neuroprotective Effects, Activation of Energy Metabolism and Regulation of Acetylcholine Esterase by OMO in D-Galactose-Induced Deficient Rats

**FIGURE 1:**
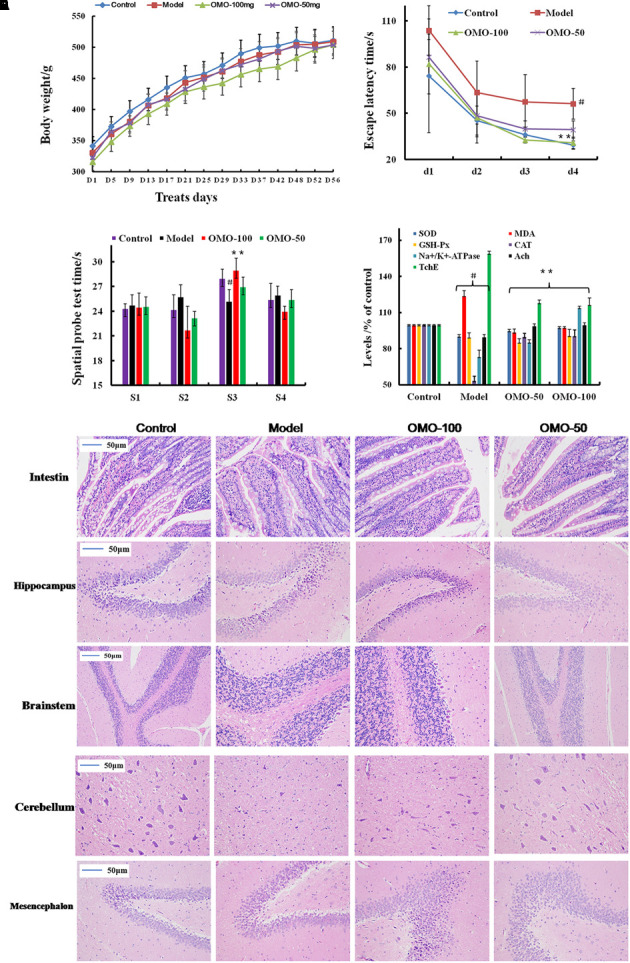
Effect of OMO in D-galactose-induced deficient rats. **(A)** Body weight changes during the treatments time. **(B)** Escape latency in the MWM. **(C)** Swimming time in the platform quadrant during the spatial probe test. **(D)** Effect on SOD, MDA, CAT, GSH-Px TchE, Ach, and Na^+^/K^+^-ATPase levels. **(E)** Histopathological changes in the intestine and brain. The graph Control, control group; Model, model group; OMO-50 mg, low-dose group that received D-galactose (100 mg/kg/d) by i.p. and gavage at a dosage of 50 mg/[kg⋅d] in OMO; OMO-100 mg, high-dose group that received D-galactose (100 mg/kg/d) i.p. and gavage at a dosage of 100 mg/[kg⋅d] in OMO. Values are represented as mean ± SD (*n* = 6) and expressed as the percentage of the control group, ^#^*p* < 0.01 vs. control group, ^∗^*p* < 0.05 vs. model group, ^∗∗^*p* < 0.01 vs. model group.

The fur of the treated animals was much smoother than that of the model group. The average weight between the treated and the model groups did not differ significantly (*p* > 0.05); the animals weighed approximately 320 g at the beginning and 500 g at the end of the experiment (**Figure 1A**).

Compared to the model group, the incubation period for each OMO-treated group was significantly shorter. The incubation period for the low-dose OMO group was (86.37 ± 11.46 s) and that for the high-dose group was (82.00 ± 19.44 s) on the 1st day. Compared to the model group, the differences were significant (*p* < 0.01). On the 4th day, the incubation period for the low-dose OMO group was (39.30 ± 5.63 s) and that for the high-dose group was 30.74 ± 3.69 s; the differences were significant as compared to the model group (*p* < 0.01; **Figure 1B**). These results demonstrated that OMO administration could ameliorate D-galactose-induced learning and memory dysfunction in rats.

Probe test results did not reveal any significant differences (*p* > 0.05) among the groups with respect to total swimming distance or speed. The swimming time of the control group in the NW quadrant (28.00 ± 0.81 s) was significantly longer than that in the other three quadrants (24.36 ± 0.40, 24.21 ± 1.33, and 25.43 ± 1.465 s; *p* < 0.01). The swimming time in the NW quadrant of the model group was 25.23 ± 1.04 s, which was significantly shorter than the control group (*p* < 0.01), suggesting that the rats remembered the location of the placement of the platform. The swimming durations of the low- and high-dose OMO groups were 27.13 ± 0.85 and 29.00 ± 1.08 s, respectively, which were significantly longer than the model group. Compared to the model group, the differences were significant (*p* < 0.01; **Figure 1C**).

As shown in **Figure 1D**, the SOD levels in the low- and high-dose OMO groups were 95.05 ± 1.21, and 97.70 ± 1.43 (% of control), respectively, as compared to 90.45 ± 2.17 in the model groups. The differences were significant as compared to the model group (*p* < 0.05). In addition, the levels of GSH-Px and CAT showed similar trends, while that of the MDA showed opposite trends, suggesting that OMO encouraged SOD, MDA, CAT, and inhibited MDA production, thereby indicating that OMO administration can enhance the antioxidative activities in the D-galactose-induced deficient rats.

To evaluate the protective efficacy of OMO on the energy metabolism in D-galactose-treated rats, we measured the Na^+^/K^+^-ATPase levels in brain tissue. As shown in **Figure 1D**, the levels of Na^+^/K^+^-ATPase were significantly lower in the model group (73.53 ± 5.17% of control) as compared to the control group (*p* < 0.05). The levels of all the OMO-treated groups (90.15 ± 2.08 for low-dose, 90.83 ± 1.64 for high-dose) were increased significantly, and the differences were significantly different as compared to the model group (*p* < 0.05). These levels were based on the concentration-dependent activities; however, the specific underlying mechanism necessitates further studies.

Cholinergic system damage and abnormal ACh levels are observed in AD patients. The results are illustrated in **Figure 1D**. As compared to the model group (89.85 ± 1.93% of control), after treatment with different concentrations (50 and 100mg/[kg⋅d]) of OMO, the ACh levels of the model rats increased significantly to 99.22 ± 1.49 and 99.98 ± 1.52, respectively, (*p* < 0.05). The AchE decreased to 118.39 ± 1.93 and 116.70 ± 5.47, respectively. These differences were significant (*p* < 0.05) as compared to the model group (159.37 ± 4.15).

The HE staining of the small intestinal tissues revealed crypt atrophy, distortion, and surface irregularity in the model group D-galactose-induced deficient rats, while those changes in the OMO-treated groups were improved (**Figure 1E**, intestine). Moreover, the staining did not demonstrate any remarkable neuronal abnormalities in the hippocampus of the rats in the control group. The pyramidal cells in the CA1 region were arranged precisely and tightly, and no cell loss was observed. Additionally, in the control group, the cells were round and intact with stained clear, dark blue nuclei (**Figure 1E**). However, noticeable damage in the hippocampus was observed in the model groups by histopathology. The pyramidal layered structure was disintegrated, and the neuronal loss was found in the CA1 region. Neurons with pyknotic nuclei and shrunken or irregular shape were also observed. These abnormalities were attenuated by the treatment with OMO. The cells in the OMO-treated groups exhibited superior cell morphology and were more in number than those in the untreated groups, especially those in the OMO-100 treated group were superior to the control group. Together, these results demonstrated that the OMO administration could ameliorate the D-galactose-induced deficient rats.

#### Changes in Gut Microbiota after OMO Administration

**FIGURE 2:**
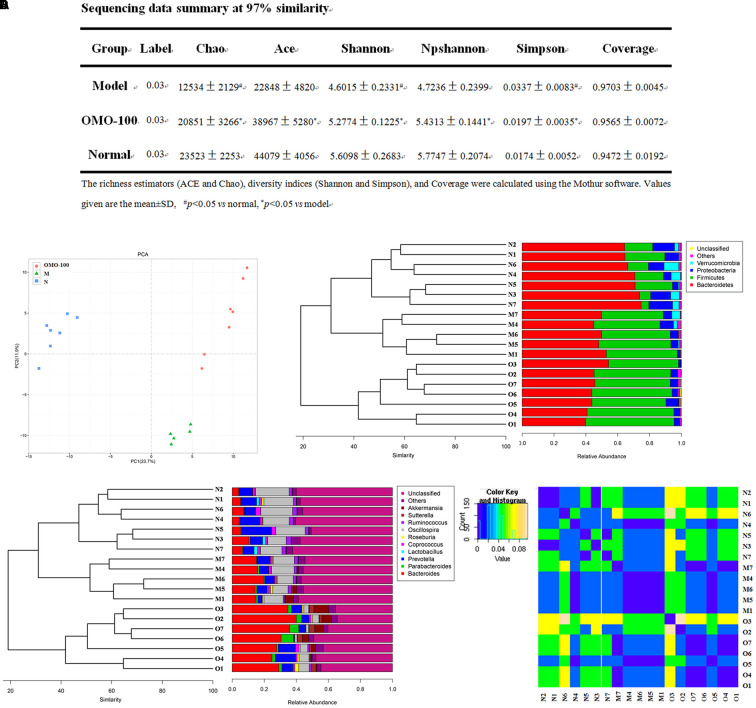
Effects of OMO on gut microbiota in D-galactose-induced deficient rats. **(A)** Sequencing data summary at 97% similarity. **(B)** Results of PCA. **(C)** Classification and abundance of cecal contents at the phylum level. **(D)** Classification and abundance of cecal contents at the genus level, and the beta diversity. The graph N is normal group; M is model group; O is OMO-100 mg, high-dose group that received D-galactose (100 mg/kg/d) i.p., and gavage at a dosage of 100 mg/[kg⋅d] in OMO. Values are the means of six independent experiments (*n* ≥ 5).

Operational taxonomic unit (OTU) abundance and taxonomic profiles were analyzed as shown in **Figure 2**. The values of Chao1, ACE, Shannon, and npShannon were reduced, and that of Simpson was increased significantly in the D-galactose-induced group than the normal group (*p* < 0.05, **Figure 2A**). After treatment with 100 mg/kg/d OMO, the values of Chao1, ACE, Shannon, npShannon, and Simpson were improved to resemble the normal (**Figure 2A**). All the treated groups could be clustered using the principal component analysis (PCA) (**Figure 2B**); Bray–Curtis distance was shown at the phylum level in **Figure 2C** (*Verrucomicrotia*, *Proteobacteria*, *Firmicutes*, and *Bacteroidetes*), genus level in **Figure 2D** (left) (*Prevotella*, *Oscillospira*, *Lactobacillus*, *Bacteroides*, *Parabacteroides*, *Sutterella*, *Akkermansia*), and beta diversity at the genus level in **Figure 2D** (right). Our results indicated that OMO administration could maintain the abundance of gut microbiota in D-galactose-induced deficient rats, although additional studies are warranted.

### Effects of OMO on Aβ_1-42_-Induced Deficient Rats

#### Indexes Improvements by OMO Administration

**FIGURE 3:**
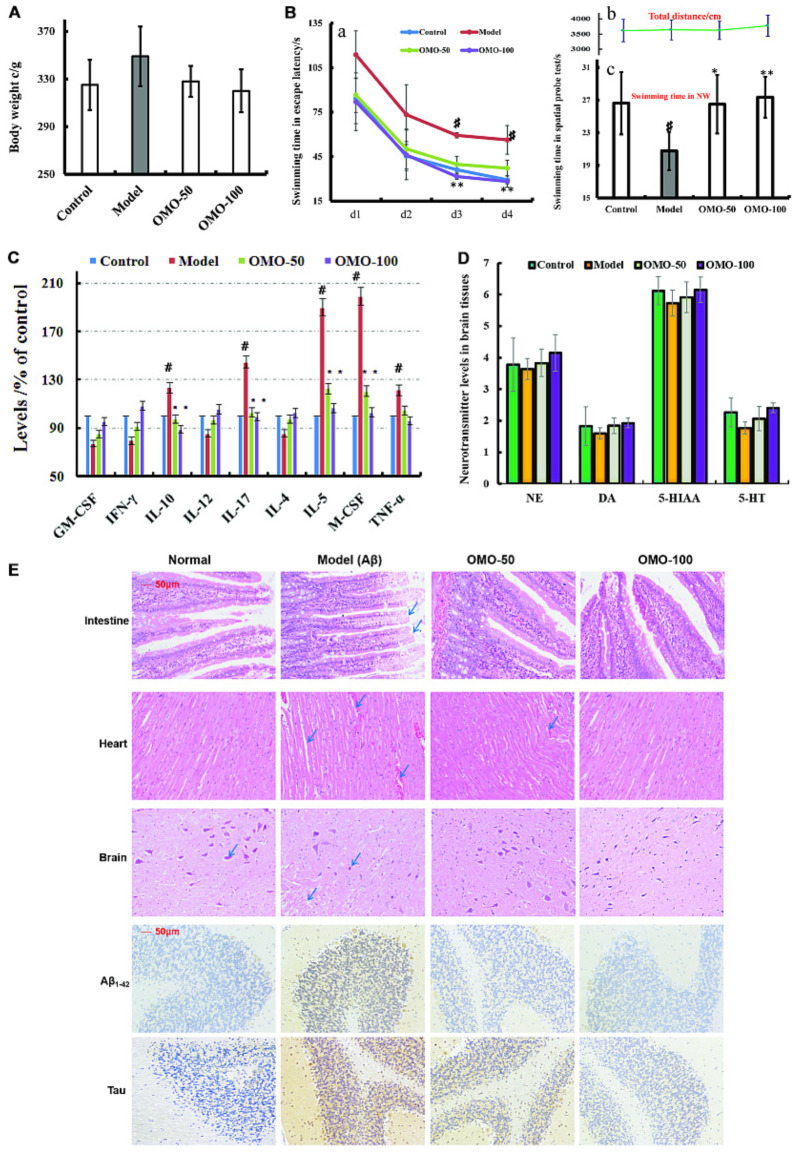
Effect of OMO in Aβ_1-42_-induced deficient rats. **(A)** Body weight changes during the treatments time. **(B-a)** Escape latency in the MWM. **(B-b)** Swimming distance. **(B-c)** Swimming time in the platform quadrant during the spatial probe test. **(C)** Level of cytokines GM-CSF, TNF-γ, 1L-10, IL-12, 1L-17α, 1L-4, TNF-α, and VGEF-α in the serum. **(D)** Levels of monoamine neurotransmitters (NE, DA, 5-HT, and 5-HIAA) in the brain tissue. **(E)** Histopathological changes in the intestine, heart, and brain, and the expressions of Aβ_1-42_ and Tau proteins in brain tissues by immunohistochemistry. The graph Control, control group; Model, model group; OMO-50 mg, low-dose group that received D-galactose (100 mg/kg/d) i.p. and gavage at a dosage of 50 mg/[kg⋅d] in OMO; OMO-100 mg, high-dose group that received D-galactose (100 mg/kg/d) i.p. and gavage at a dosage of 100 mg/[kg⋅d] in OMO. Values are represented as mean ± SD (*n* = 6) and expressed as the percentage of the control group, ^#^*p* < 0.01 vs. control group, ^∗^*p* < 0.05 vs. model group, ^∗∗^*p* < 0.01 vs. model group.

The fur of the treated animals was much smoother than that of the model group. The average weight of model groups was found to be higher than that of the control group (*p* < 0.05), and we found that the rats in the model group present constipation and swollen belly. Moreover, the weight records of OMO-treated groups did not alter significantly (*p* > 0.05) as compared to the control group (**Figure 3A**).

Compared to the model group, the incubation period for each OMO-treated group was significantly shorter. The incubation period for the low-dose OMO group was 86.49 ± 11.64 s, while that for the high-dose group was 82.06 ± 19.44 s on the 1st day. Compared to the model group (113.75 ± 16.11 s), the differences were significant (*p* < 0.01). On the 4th day, the incubation period of the low-dose OMO group was 37.19 ± 5.36 s, and that for the high-dose group was 28.27 ± 3.96 s; the differences were significant as compared to the model group (56.29 ± 9.69 s, *p* < 0.01; **Figure 3Ba**). These results showed that OMO administration could ameliorate the Aβ_1-42_-induced learning and memory dysfunction in rats.

Probe test results showed no significant differences (*p* > 0.05) among the groups with respect to the total swimming distance or speed. The swimming time of the control group in the NW quadrant (26.63 ± 3.83 s) was significantly longer than that in the other three quadrants (*p* < 0.01). The swimming time in the NW quadrant of the model group was 20.77 ± 2.36 s, which was significantly shorter than the control group (*p* < 0.01), suggesting that the rats remembered the location of the platform. The swimming time of the low- and high-dose OMO groups were 26.50 ± 3.59 and 27.36 ± 2.51 s, which were significantly longer than the model group. Compared to the model group, the differences were significant (*p* < 0.01), as shown in **Figure 3Bc**. The swimming distances did not differ among all groups (**Figure 3Bb**).

All the cytokines’ levels in the serum of Aβ_1-42_-induced group deviated from the normal; GM-CSF, TNF-γ, 1L-10, IL-12, 1L-17α, 1L-4, TNF-α, and VGEF were secreted significantly different (*p* < 0.05 or *p* < 0.01; **Figure 3C**). When treated with OMO, all these cytokines were strikingly recovered close to the baseline level (**Figure 3C**), thereby indicating that OMO administration can improve the inflammatory environment.

The monoamine neurotransmitter levels in right brain tissue were dissected from 4 rats in each group, according to the methods described previously (Chen et al., 2014b). **Figure 3D** showed that the levels of norepinephrine (NE), dopamine (DA), 5-hydroxytryptamine (5-HT), and 5-hydroxyindole acetic acid (5-HIAA) were reduced in Aβ_1-42_-induced groups as compared to the control group, and the OMO administration can promote the secretion of some monoamine neurotransmitters (NE, DA, 5-HT, and 5-HIAA) in a concentration-dependent manner (**Figure 3D**).

The HE staining of small intestinal tissues revealed crypt branching, atrophy, distortion, and surface irregularity in the model group Aβ_1-42_-induced deficient rats, while those changes in the OMO-treated groups were improved (**Figure 3E**, intestine). The injury to the atrial tissues in the Aβ_1-42_-induced group was more severe than that in the normal and OMO-treated groups. Furthermore, the HE staining did not reveal any remarkable neuronal abnormalities in the hippocampus of rats in the control group (**Figure 3E**). However, the obvious hippocampal histopathological damage was observed in the model groups. The pyramidal layered structure was disintegrated, and neuronal loss was found in the CA1 region. These abnormalities were attenuated by OMO treatment. The cells in OMO-treated groups exhibited better cell morphology and were more in number than those in the untreated groups, especially those in the OMO-100 treated group were superior to the control group. Compared to the normal group, the proportion of Aβ_1-42_ and Tau-positive cells in rats in the model group was significantly higher than that in the normal group (*p* < 0.05), while the OMO administration down-regulated the expression of Aβ_1-42_ and Tau proteins (**Figure 3E**).

#### The Gut Structure of the Microbiota Was Altered Significantly by OMO

**FIGURE 4:**
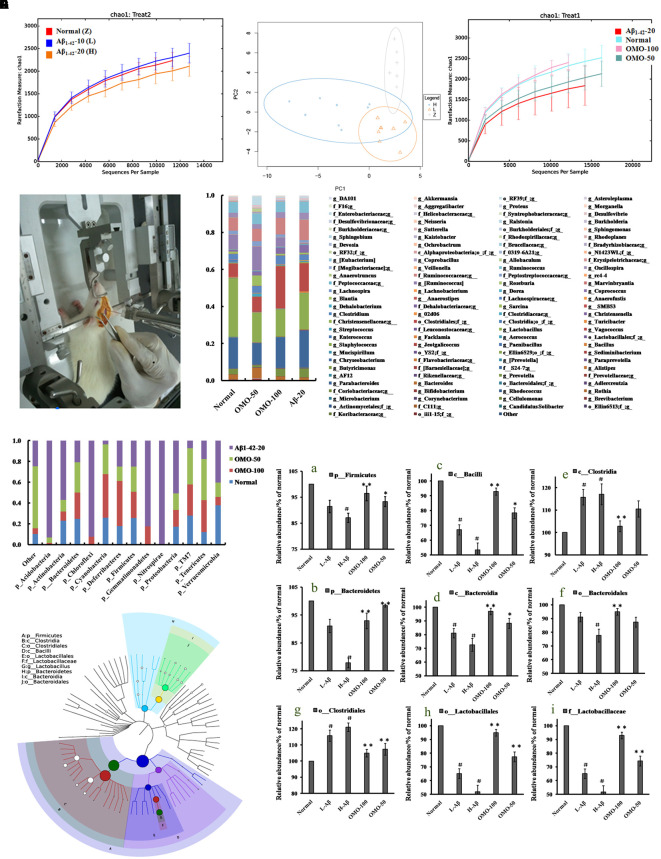
Effects of OMO on gut microbiota in Aβ_1-42_-induced deficient rats. **(A)** Rarefaction curve and **(B)** PCA results of different concentrations of Aβ_1-42_-induced groups, H (20 μg of Aβ_1-42_), L (20 μg of Aβ_1-42_), Z (normal rats with vehicle). **(C)** Rarefaction curve. **(D)** Operation schematic diagram. **(E)** Classification and abundance of cecal contents at the phylum level. **(F)** Classification and abundance of cecal contents at the genus level. **(G)** The dominant species classification tree. **(H)** The relative abundance of the dominant microorganism. Values are the means of six independent experiments.

OTU abundance and taxonomic profiles were analyzed as shown in **Figure 4**. Compared to the normal group, the diversity of the microbial communities in Aβ_1-42_-induced (injected 10 or 20 μg fibrillar state of Aβ_1-42_ into the CA1 region, **Figure 4D**) group was reduced and negatively related with the doses of Aβ_1-42_ (**Figure 4A**); All the Aβ_1-42_-induced groups were clustered as expected using PCA (**Figure 4B**), which indicated that the Aβ not only influences the brain but also changes the gut microbiota. After treatment with 50 mg/(kg⋅d) or 100 mg/(kg⋅d) OMO, the diversity of the microbial communities was improved similarly as that of the normal (**Figure 4C**).

Analysis revealed the difference of taxonomic abundance between different groups (**Figure 4E**). Some bacteria in the fecal samples changed considerably at the phylum level: for instance, *Verrucomicrotia*, *Proteobacteria*, *Firmicutes*, and *Bacteroidetes*. Moreover, at the genus level, the Aβ_1-42_-induced rats exhibited the enrichment of potentially proinflammatory microbes, such as *Corynebacterium, Staphylococcus, Ruminococcus, Roseburia, Dorea*, and *Sutterella*, and the reduction of potentially anti-inflammatory microbes, such as *Bacteroides, Bifidobacterium, Prevotella, Parabacteroides, Coprococcus, Desulfovibrio*, and *Lactobacillus*, in comparison with the normal group (**Figure 4F**). However, the treatment with the OMO exhibited a reduction in proinflammatory microbes and enrichment of anti-inflammatory microbes. Thus, our results indicated that the OMO administration had the potential to regulate the structure of the gut microbiota.

We also constructed and visualized a taxonomic tree of the predominant taxa (**Figure 4G**), which showed that *Firmicutes*, *Bacteroidetes*, *Clostridia*, *Bacteroidia*, *Bacilli*, *Clostridiales*, *Lactobacillales*, *Bacteroidales*, *Lactobacillaceae*, and *Lactobacillus* were the predominant taxa. The altered details of the predominant taxa (**Figure 4H**) showed that the abundance of *Clostridia* and *Clostridiales* in Aβ_1-42_-induced groups was increased sharply, while the aforementioned taxa as *Firmicutes*, *Bacteroidetes*, *Bacteroidia*, *Bacilli*, *Lactobacillales*, *Bacteroidales*, *Lactobacillaceae*, and *Lactobacillus* were reduced (*p* < 0.05 vs. normal group). On the other hand, the OMO-treated groups can reverse those changes, especially the probiotic *Lactobacillus* increased obviously, which indicated that OMO administration might have a prebiotic role in intestinal dysbacteriosis in AD animals as induced by Aβ_1-42_.

### Prebiotic Effect of OMO on TNBS-Induced Mice

#### The Tissue Damages and Inflammation Induced by TNBS Combined Antibiotics Were Relieved

**FIGURE 5:**
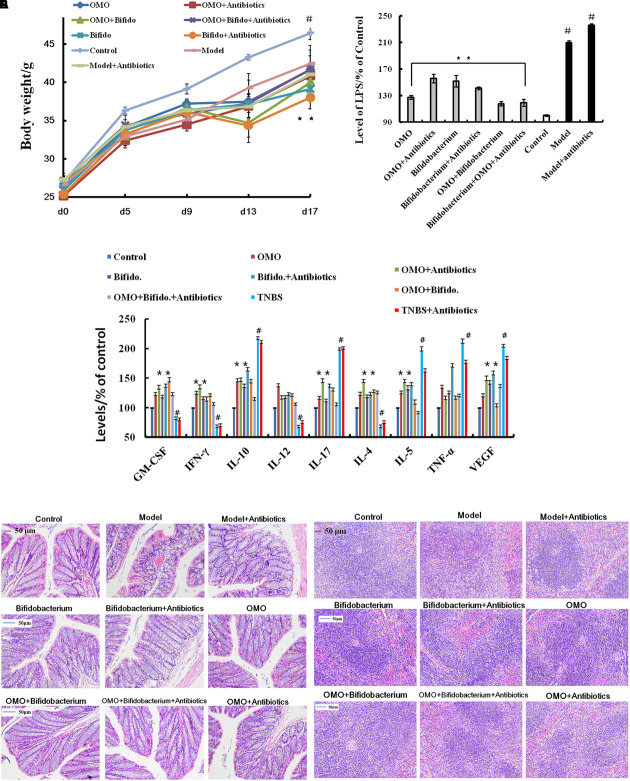
OMO improves the pathological parameters of the high-dose broad spectrum antibiotics and TNBS-induced inflammatory bowel disease (IBD) mice. **(A)** Body weight changes. **(B)** Levels of LPS in serum. **(C)** The levels of cytokines (GM-CSF, TNF-γ, 1L-10, IL-12, 1L-17α, 1L-4, TNF-α, and VGEF-α) in serum. **(D)** The histopathological changes in colon. **(E)** The histopathological changes in spleen. Control is the normal group; model is the TNBS-induced group; model and high-dose antibiotics, HEP3 (100 mg/kg/d), *Bifidobacterium*, HEP3 and high-dose antibiotics, HEP3 and *Bifidobacterium*, *Bifidobacterium* and high-dose antibiotics, HEP3, *Bifidobacterium*, and high-dose antibiotics.

**FIGURE 6:**
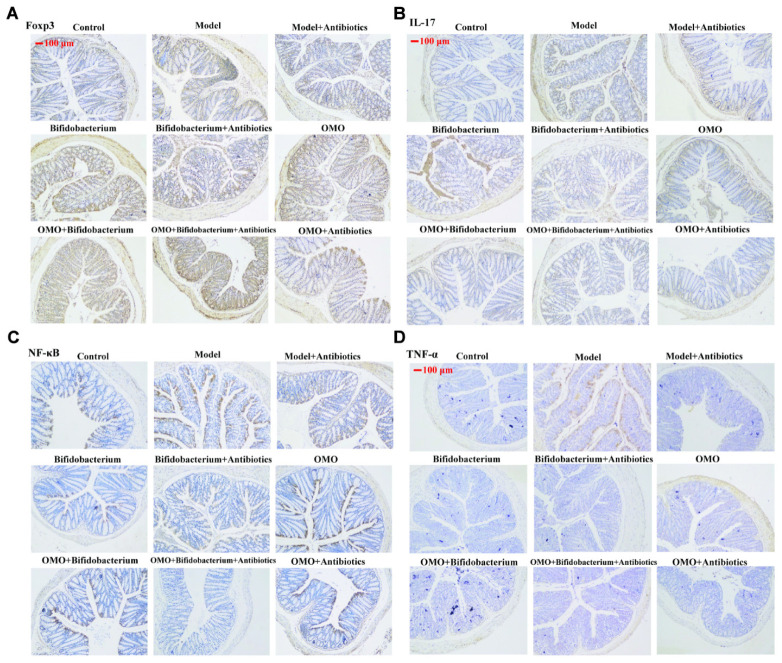
Immunohistochemistry staining of Foxp3 **(A)**, IL-17 **(B)**, NF-κB p65 **(C)**, and TNF-α **(D)** in the colons of different experimental groups in high-dose broad spectrum antibiotics and TNBS-induced IBD mice after treatment with OMO.

In order to ensure the prebiotic role of OMO, we established an IBD mice model after a broad spectrum antibiotics treatment. Compared to the control group, post treatment with TNBS by enema, a majority of the mice presented diarrhea and the weight gain declined relatively (**Figure 5A**). All the cytokines’ levels were deviated from the normal, as some anti-inflammatory cytokines of GM-CSF, TNF-γ, 1L-10, IL-12, 1L-17α, 1L-4, TNF-α, and VGEF were secreted differently (*p* < 0.05 or *p* < 0.01; **Figure 5C**). Simultaneously, we found that the content of lipopolysaccharide (LPS) (**Figure 5B**) was higher than that in the control group; the colon tissues (**Figure 5D**) and splenic tissues (**Figure 5E**) were severely damaged. Also, immunohistochemistry staining showed that the expressions of Foxp3 (**Figure 6A**), IL-17 (**Figure 6B**), NF-κB (**Figure 6C**), and TNF-α (**Figure 6D**) deviated from the control, especially the additional broad spectrum and overdose antibiotics groups. After treatment with OMO, all the deviated parameters returned to the baselines, especially the OMO + *Bifidobacterium*-treated group (**Figures 5**, **6**). Cumulatively, our results suggested that OMO and *Bifidobacterium* exert anti-inflammatory effects in IBD, synergistically; however, the underlying mechanism needs further studies.

#### Promotion of the Engraftment Ability of *Bifidobacterium*

**FIGURE 7:**
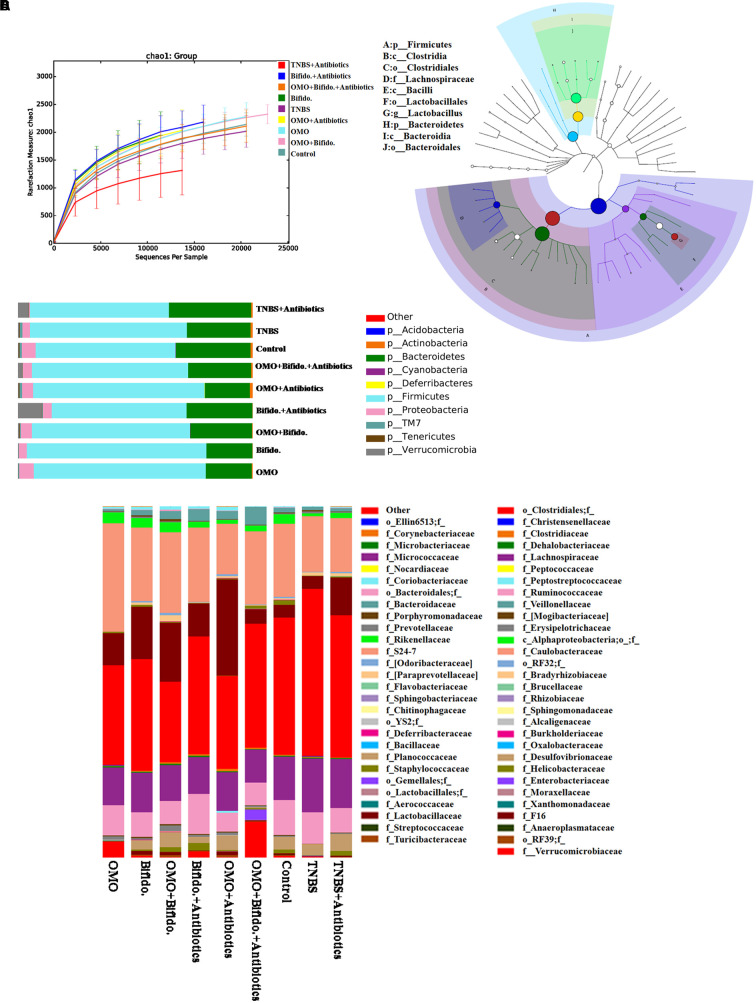
Effects of OMO on the microbiota of cecal contents in high-dose broad spectrum antibiotics and TNBS-induced IBD mice. **(A)** The graph represents the rarefaction curve. **(B)** The dominant species classification tree. **(C)** The classification and abundance of cecal contents at the phylum level. **(D)** The classification and abundance of cecal contents at the family level. Values are the means of six independent experiments.

To clarify the synergistical action between OMO and *Bifidobacterium*, OTU abundance and taxonomic profiles were analyzed as shown in **Figure 7**. Compared to the normal group, the diversity of the microbial community in TNBS and TNBS and antibiotics-induced groups was reduced (**Figure 7A**). OMO can ameliorate this dysbacteriosis; the bacterial compositions at the phylum (**Figure 7C**) and family level (**Figure 7D**) encompassed *Verrucomicrotia*, *Proteobacteria*, *Firmicutes*, *Bacteroidetes*, *Lactobacillaceae*, and *Lachnospiraceae*. Our results showed that the relative abundance of *Bifidobacterium* was increased remarkably (*p* < 0.05, **Figure 5**), and the other probiotics *Lactobacillaceae* were also abundant in the microbial community with stable structures. We also constructed and visualized a taxonomic tree of the predominant taxa (**Figure 7B**), which showed that the *Firmicutes*, *Bacteroidetes*, *Clostridia*, *Bacteroidia*, *Bacilli*, *Clostridiales*, *Lactobacillales*, *Bacteroidales*, *Lactobacillaceae*, and *Lactobacillus* were the predominant taxa.

### Influence of Aβ Levels on Microbiota-Gut-Brain Axis

In order to explore whether Aβ can influence the gut microbiota by targeting the microbiota-gut-brain axis, we injected 10 and 20 μg Aβ_1-42_ into the CA1 region, respectively. Then, the fecal samples were collected once a week and the last 4 weeks, followed by microbiome analysis using the 16S rRNA genes. We also monitored the transcriptome of the small intestine and brain tissues at the 5th week after injection of Aβ_1-42_.

#### Dynamic Variations of Gut Microbiota and KEGG Pathway Analysis in the Aβ_1-42_-Induced Deficient Rats

**FIGURE 8:**
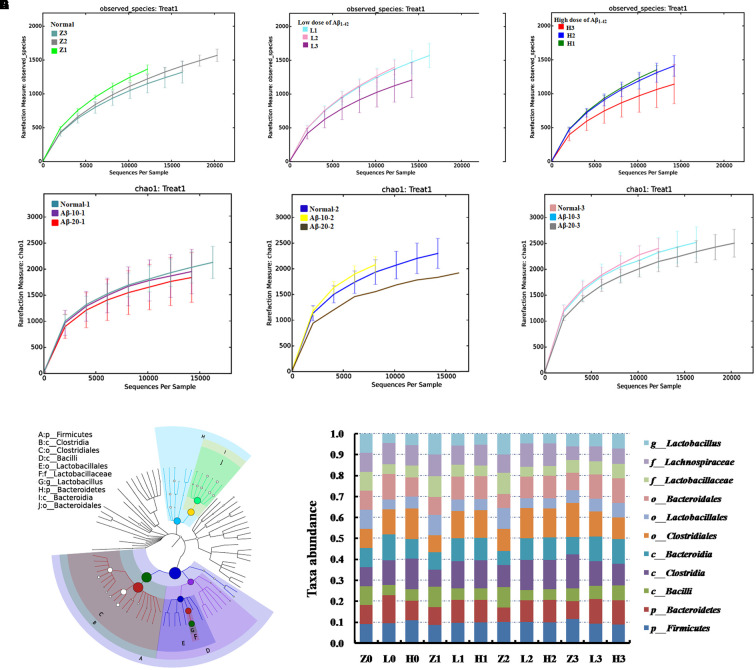
Effects of Aβ_1-42_ on gut microbiota in rats. **(A–F)** The rarefaction curve of different concentrations and durations in Aβ_1-42_-induced groups, H (20 μg of Aβ_1-42_), L (20 μg of Aβ_1-42_), Z (normal rats with vehicle); H1, H2, and H3 (or L1, L2, L3) is the treatment time after injection of Aβ_1-42_ at 2nd, 3rd, and 4th weeks; **(G)** is the dominant species classification tree; **(H)** is the relative abundance of the dominant microorganism. Values represent the means of six independent experiments.

The dynamic variations of gut microbiota showed that the diversity in the Aβ_1-42_-induced microbial community (injected 10 or 20 μg fibrillar state of Aβ_1-42_ into the CA1 region) group was reduced in the 4th week (L3, H3) than that in the 2nd and 3rd week (L1, L2 and H1, H2), as shown in **Figures 8B,C**, thereby indicating that such a diversity was reduced with the progression of the disease. **Figures 8D–F** showed that the diversity of the microbial community changed with the levels of Aβ_1-42_, which revealed that the levels of Aβ_1-42_ in the brain influenced the composition of the gut microbiota significantly. We also constructed and visualized a taxonomic tree of the predominant taxa (**Figure 8G**), which displayed that the *Firmicutes*, *Bacteroidetes*, *Clostridia*, *Bacteroidia*, *Bacilli*, *Clostridiales*, *Lactobacillales*, *Bacteroidales*, *Lactobacillaceae*, and *Lactobacillus* were the predominant taxa. The changes in the details of predominant taxa (**Figure 8H**), along with the abundance of *Lactobacillaceae*, were negatively correlated with the dose of Aβ_1-42_.

**FIGURE 9:**
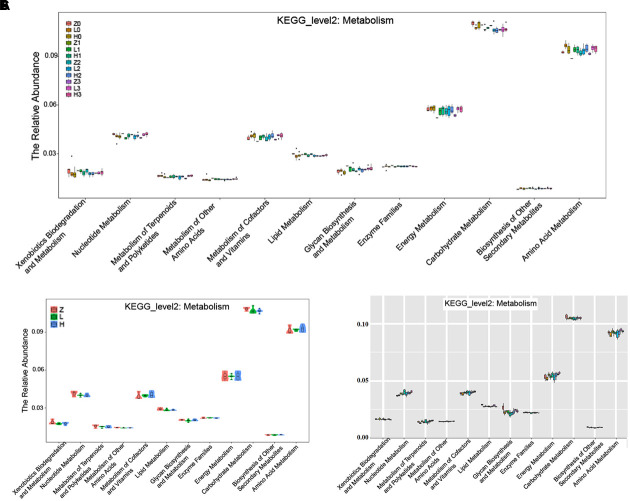
The KEGG pathway enrichment of gut microbiota in the metabolism system. **(A)** The dynamic variations in the 2nd, 3rd, and 4th week after injection of Aβ_1-42_ in rats. **(B)** The 4th week after injection of Aβ_1-42_ in rats. **(C)** The high dose broad spectrum antibiotics and TNBS-induced IBD mice.

The KEGG pathway analysis showed that the metabolism of xenobiotics biodegradation, nucleotide metabolism, metabolism of terpenoids and polyketides, metabolism of other amino acids, metabolism of cofactors and vitamins, lipid metabolism, glycan biosynthesis and metabolism, enzyme families, energy metabolism, carbohydrate metabolism, biosynthesis of other secondary metabolites, and amino acid metabolism were altered according to the gut microbiota in the two AD-like rodent models. Additionally, many of these metabolisms were improved by the administration of OMO (**Figure 9A**) showed the dynamic variations in the 2nd, 3rd, and 4th week after injection of Aβ_1-42_. **Figure 9B** represents the 5th week after injection of Aβ_1-42_, all of which showed that the metabolism of gut microbiota was influenced by the levels of Aβ_1-42_ in hippocampus.

#### Transcriptome Analysis in Small Intestine and Brain in Deficient Rats Post Aβ_1-42_ Injection

##### Small intestine transcriptome analysis

**FIGURE 10:**
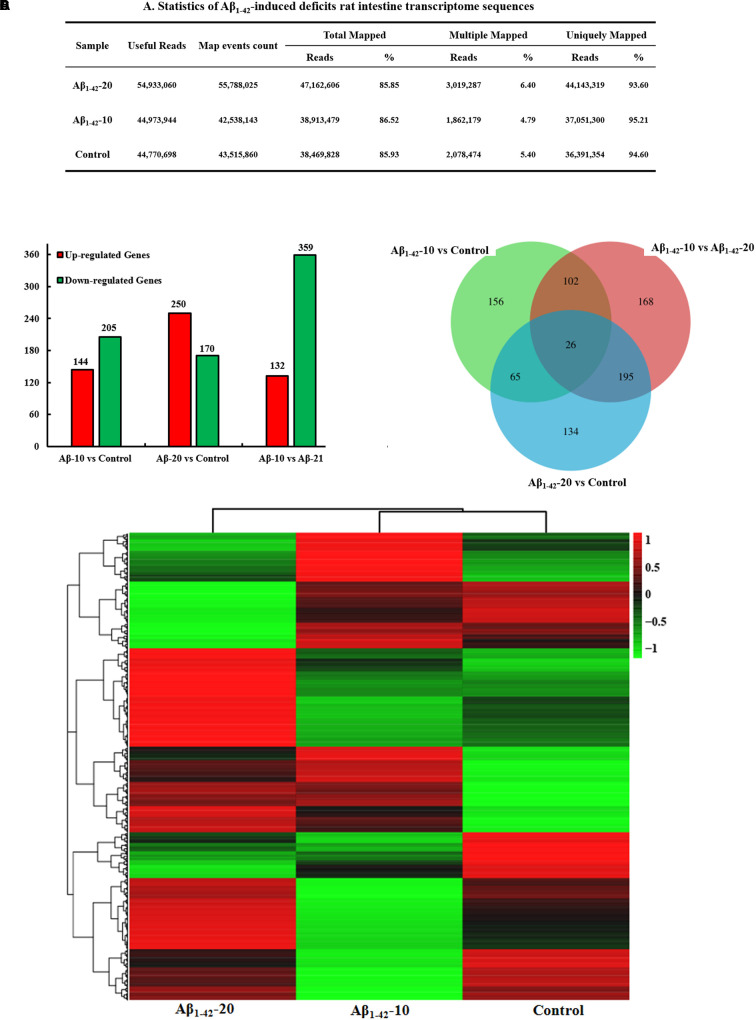
Small intestine transcriptome analysis in the deficit rats by injected Aβ_1-42_. **(A)** The statistics of transcriptome sequences. **(B)** DEGs in the small intestine at different concentrations of Aβ_1-42_. **(C)** Venn diagram of DEGs. **(D)** The heat map of the relative expressions of DEGs in all three groups.

After injection of Aβ_1-42_ for 4 weeks, the rats were sacrificed, and the intestinal tissues were dissected and frozen in liquid nitrogen for RNA extraction and high-throughput RNA-sequencing. To obtain an overview of the gene expression profile of the intestine in AD model rats, three cDNA samples were generated from each group, mixed, and subjected to sequencing by the Illumina NextSeq 500 platform. Approximately 45,323,472, 55,358,634, and 45,134,740 raw reads with a length of 2 × 150 bp were generated for the Aβ_1-42_-20, Aβ_1-42_-10, and control group samples, respectively. After stringent quality assessment and data filtering, 44,973,944, 54,933,060, and 44,770,698 clean paired-end sequence reads with a Q20 percentage (those with a base quality >20) over 99% were obtained from the differently treated samples, respectively. Of all the reads, approximately 86.0% were mapped to the rat genome. Based on the normalized data, the expression of 21933 genes was detected (**Figure 10A**), and the relative expressions of DEGs in all the three treated groups (Aβ_1-42_-10, Aβ_1-42_-20, and control) were shown in **Table 1**.

**Table 1:** Differentially expressed genes (DEGs) in the small intestine of deficient rat injected at different concentration of Aβ_1-42_.

Genes	FoldChange		log2FoldChange		Up–down	*p*-value	
	Normal/Aβ-10	Normal/Aβ-20	Normal/Aβ-10	Normal/Aβ-20		Normal/Aβ-10	Normal/Aβ-20
Slc5a4b	0.09	0.27	-3.47	-1.86	Down	0.000000	0.000494
Nts	0.41	4.57	-1.27	2.19	Down	0.002207	0.000056
Fkbp5	0.27	2.81	-1.90	1.49	Down	0.000004	0.000132
Cubn	0.40	2.81	-1.33	1.49	Down	0.000323	0.000132
Defa8	0.03	0.08	-5.11	-3.66	Down	0.000000	0.000000
AABR07000398.1	0.45	3.21	-1.17	1.68	Down	0.000995	0.000002
LOC257642	0.45	4.12	-1.15	2.04	Down	0.032226	0.012780
Hpse	5.37	0.39	2.43	-1.36	Up	0.000050	0.002196
Nop10	2.12	10.70	1.09	3.42	Down	0.032836	0.000000
Arhgdib	2.22	0.49	1.15	-1.03	Up	0.006915	0.007868
AABR07065789.1	2.13	7.85	1.09	2.97	Down	0.026618	0.000001
Reg3g	2.72	0.35	1.44	-1.52	Up	0.000232	0.000032
Rps24	4.88	66.16	2.29	6.05	Down	0.000000	0.000000
Pou2af1	2.14	0.36	1.10	-1.47	Up	0.048293	0.001217
Adh6	2.32	0.14	1.21	-2.88	Up	0.004548	0.000000
RGD1311933	2.24	5.12	1.16	2.36	Down	0.006467	0.000000
Rps27	2.08	19.07	1.05	4.25	Down	0.003563	0.000000
Wfdc21	2.46	0.45	1.30	-1.15	Up	0.002445	0.002823
Rps27a-ps1	3.22	6.84	1.69	2.77	Down	0.000020	0.000000
Ccl21	3.20	0.30	1.68	-1.73	Up	0.001820	0.000047
Pdx1	3.20	0.49	1.68	-1.03	Up	0.007298	0.037669
AABR07051670.1	7.56	0.36	2.92	-1.49	Up	0.000008	0.000992
Igkv5-48	30.08	0.32	4.91	-1.65	Up	0.000000	0.000302
AABR07051684.1	23.10	0.27	4.53	-1.91	Up	0.001867	0.003488
AABR07065768.3	23.64	0.11	4.56	-3.25	Up	0.032367	0.000002
Akp3	13.94	0.13	3.80	-2.93	Up	0.000000	0.000000

The general chi-squared test was used for the selection of significant DEGs. Based on the criteria of twofold or greater change and *Q* of *p* < 0.05, 349 unigenes were identified as significant DEGs between Aβ_1-42_-10 and control group samples and 420 unigenes between Aβ_1-42_-20 and control group samples (**Figure 10B**). To elucidate the DEGs in different contents of Aβ_1-42_-induced groups, we used the gene expression profiling. As illustrated in the Venn diagram (**Figure 10C**), the number of genes, as well as the relationships among the overlap between the different groups, were shown in **Figures 10B,D**, indicating that Aβ_1-42_ level in the brain can influence the transcriptome of the intestine.

To gain insights into the physiological processes regulated by the different Aβ_1-42_ levels and identify the processes enriched in significant DEGs, we subjected significant DEGs to GO term enrichment analysis and KEGG pathway enrichment, a tool developed to represent the common and basic biological information in the annotation. The GO term enrichment results showed that the immune system, extracellular environment, and antigen reaction (Supplementary Tables S1-1–S1-3) altered in different Aβ_1-42_ levels treated groups, which indicated that the inflammatory response of the gut is activated, the variations in the gut microbiota induced by Aβ_1-42_ primarily influences the immune or inflammatory response in the gut.

**FIGURE 11:**
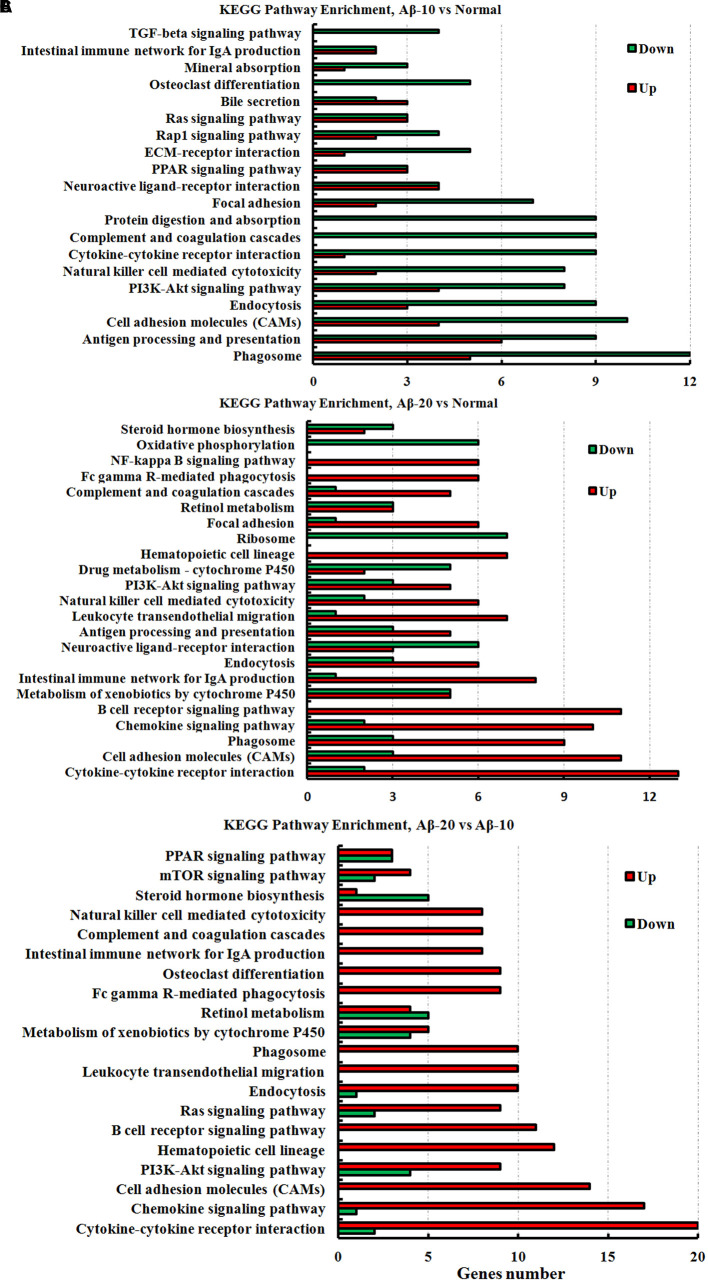
The KEGG pathway enrichment of DEGs in the small intestine transcriptome in deficient rats induced by Aβ_1-42_. **(A)** The group of Aβ-10 vs. the Normal; **(B)** the group of Aβ-20 vs. the Normal; **(C)** the group of Aβ-20 vs. the Aβ-10.

The KEGG pathway results showed that the DEGs were mainly enriched in phagosome, antigen processing, and presentation, cell adhesion molecules (CAMs), PI3K-Akt signaling pathway, cytokine-cytokine receptor interaction, PPAR signaling pathway, ECM-receptor interaction, B cell receptor signaling pathway, and chemokine signaling pathway (**Figure 11**, and more details were showed in Supplementary Tables S2-1–S2-3), thereby indicating that Aβ_1-42_ levels in the brain can influence the intestinal functions.

##### Brain transcriptome analysis

**FIGURE 12:**
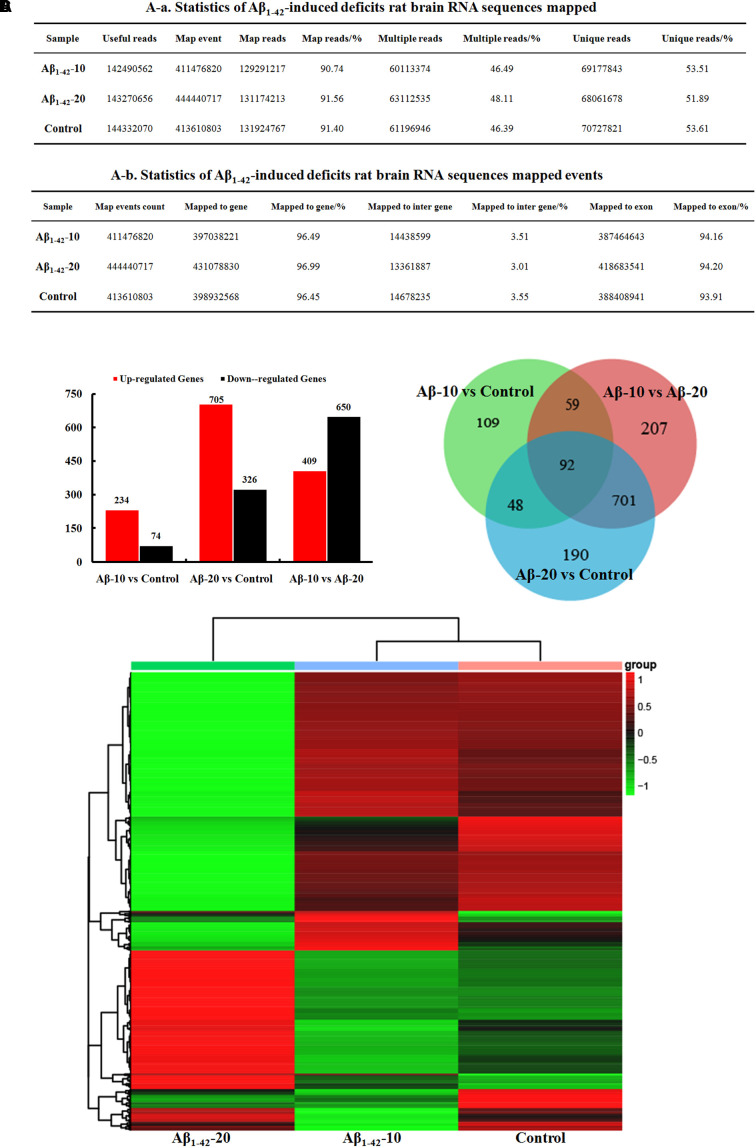
Brain transcriptome analysis in the deficit rats by injected Aβ_1-42_. **(A)** Statistics of the transcriptome sequences. **(B)** The differentially expressed genes in the brain at different concentration of Aβ_1-42_. **(C)** Venn diagram of DEGs. **(D)** The heat map of the relative expressions of DEGs in all the three groups.

After injection of Aβ_1-42_ for 4 weeks, the rats were sacrificed, and the brain tissues dissected and frozen in liquid nitrogen for RNA extraction and high-throughput RNA-sequencing. The overview of the brain gene expression profile in AD model rats was shown in **Figure 12**. The profiling analysis revealed the number of genes (**Figures 12A-a,b**), the DEGs in **Figure 12B**, Venn diagram (**Figure 12C**), as well as, the relationships among the overlap between the different groups in **Figure 12D**; the relative expressions of DEGs in all the three treated groups (Aβ_1-42_-10, Aβ_1-42_-20, and control) were shown in **Table 2**, which indicated that the Aβ_1-42_ levels could significantly influence the brain transcriptome.

**Table 2:** Differentially expressed genes in the brain of the deficient rats injected at different concentrations of Aβ_1-42._

Genes	Aβ-10		Aβ-20	
	Foldchange	*p*-value	Foldchange	*p*-value
	(Normal/Aβ-10)		(Normal/Aβ-20)	
Cbln1	10.94	0.000000	0.13	0.000105
Grm4	2.39	0.000030	0.21	0.002146
Pcp2	60.50	0.000000	0.09	0.000007
Uncx	36.00	0.000034	0.09	0.000041
Adora2a	2.31	0.000004	15.53	0.000001
Wdr66	3.13	0.000018	0.19	0.001367
Lhx5	Infinity	0.000034	0.20	0.007717
Barhl2	3.13	0.036893	0.09	0.000025
Lhx1	30.79	0.000182	0.10	0.000055
Slc6a4	10.61	0.004566	0.23	0.015495
Gabra6	107.05	0.000000	0.06	0.000000
Rgs9	2.88	0.000000	10.66	0.000012
Tph2	4.79	0.007662	0.21	0.006924
Arhgap6	2.00	0.001638	8.30	0.000186
Btg1	2.16	0.000475	0.34	0.034729
Rxrg	2.33	0.000179	15.69	0.000004
Neurod1	3.51	0.000000	0.11	0.000025
Car8	3.90	0.000000	0.12	0.000060
St14	2.78	0.017265	0.13	0.000206
Trim54	0.36	0.000003	11.11	0.000192
Grid2	2.34	0.000518	0.21	0.002420
Slc30a3	0.46	0.000006	5.77	0.000880
En2	191.37	0.000000	0.10	0.000026
Arhgef33	9.12	0.000000	0.11	0.000050
Tac1	4.23	0.000000	31.52	0.000000
Slc1a6	2.00	0.016579	0.11	0.000032
Crtam	2.24	0.000013	0.28	0.010983
Crtam	Infinity	0.000115	0.08	0.000020
Drd2	2.73	0.000002	10.76	0.000026
Cga	22.26	0.002718	0.08	0.000031
Kcnh5	0.37	0.000000	4.25	0.007422
Nyx	3.68	0.036361	0.13	0.000541
Fibcd1	2.76	0.000000	24.48	0.000000
Satb2	0.47	0.000200	43.65	0.000000
Hes3	7.22	0.008186	0.07	0.000006
Irx3	15.47	0.004015	0.14	0.001121
Adamts18	3.02	0.045565	0.11	0.000091
Il16	3.40	0.000000	0.10	0.000019
Tfap2b	3.39	0.022277	0.19	0.003907
Ppp1r17	17.05	0.000000	0.12	0.000083
Impg1	Infinity	0.009945	0.08	0.000060
Fat2	33.50	0.000000	0.10	0.000009
Irx2	9.63	0.003845	0.11	0.000157
Rgl3	3.76	0.000002	0.11	0.000045
Barhl1	21.79	0.003154	0.20	0.011641
Cdhr1	2.16	0.001501	18.13	0.000003
Glra1	3.33	0.005627	0.22	0.005136
Cbln2	0.43	0.000035	0.21	0.042611
Pax3	Infinity	0.035388	6.15	0.008361
Kcns1	0.12	0.000000	0.10	0.000187
Skor1	35.05	0.005526	0.16	0.000376
Zic1	3.08	0.000000	0.35	0.049800
Zic4	2.01	0.047534	14.65	0.000001
Cpne7	2.09	0.000020	77.78	0.000000
Emx1	0.46	0.001804	0.35	0.038103
Kcng4	2.18	0.000235	0.30	0.023002
Zfp521	3.24	0.000000	0.20	0.001811
Calb2	3.89	0.000000	0.17	0.000482
LOC688778	0.46	0.000054	51.87	0.000000
Atp2a3	4.19	0.000000	0.13	0.000074
Ptpn22	Infinity	0.035388	0.07	0.000080
Gng7	2.88	0.000000	24.88	0.000000
Asic4	2.15	0.000048	11.89	0.000008
Npas4	2.04	0.000529	13.37	0.000006
Cbln3	108.68	0.000000	0.08	0.000002
Adra1d	0.49	0.000370	4.54	0.006404
Ctxn3	3.24	0.006074	0.16	0.000811
Drd1	2.50	0.000005	35.19	0.000000
Syndig1l	3.07	0.000000	17.72	0.000001
Cdh15	2.20	0.000772	0.20	0.001638
Cnpy1	36.47	0.000030	0.11	0.000160
Robo3	0.22	0.000001	26.35	0.001324
Cyp11b2	0.23	0.000000	6.98	0.004899
Lrrc10b	2.40	0.000035	34.15	0.000000
Mab21l2	4.00	0.000280	0.17	0.000872
Slc6a5	4.53	0.003476	0.08	0.000009
Myom3	6.95	0.000135	0.33	0.049080
Svep1	2.27	0.000253	0.13	0.000107
Tll1	2.15	0.034882	0.20	0.003254
Irx1	5.63	0.001444	0.23	0.009492
Eomes	10.74	0.030867	0.07	0.000011
Tmem215	0.34	0.000002	10.90	0.000267
Spp1	3.32	0.000003	0.15	0.000348
Comp	6.67	0.000051	0.29	0.024367
Shisa8	3.49	0.000142	0.13	0.000128
Gpr6	2.45	0.000035	72.86	0.000000
Gdf10	4.42	0.001245	0.16	0.000964
Zic2	2.20	0.001201	0.21	0.002489
Dao	11.94	0.000011	0.12	0.000114
Lrp2	3.58	0.000003	0.28	0.013024
AABR07010944.1	Infinity	0.006930	0.12	0.001066
Sdc1	4.80	0.000003	0.11	0.000046

**FIGURE 13:**
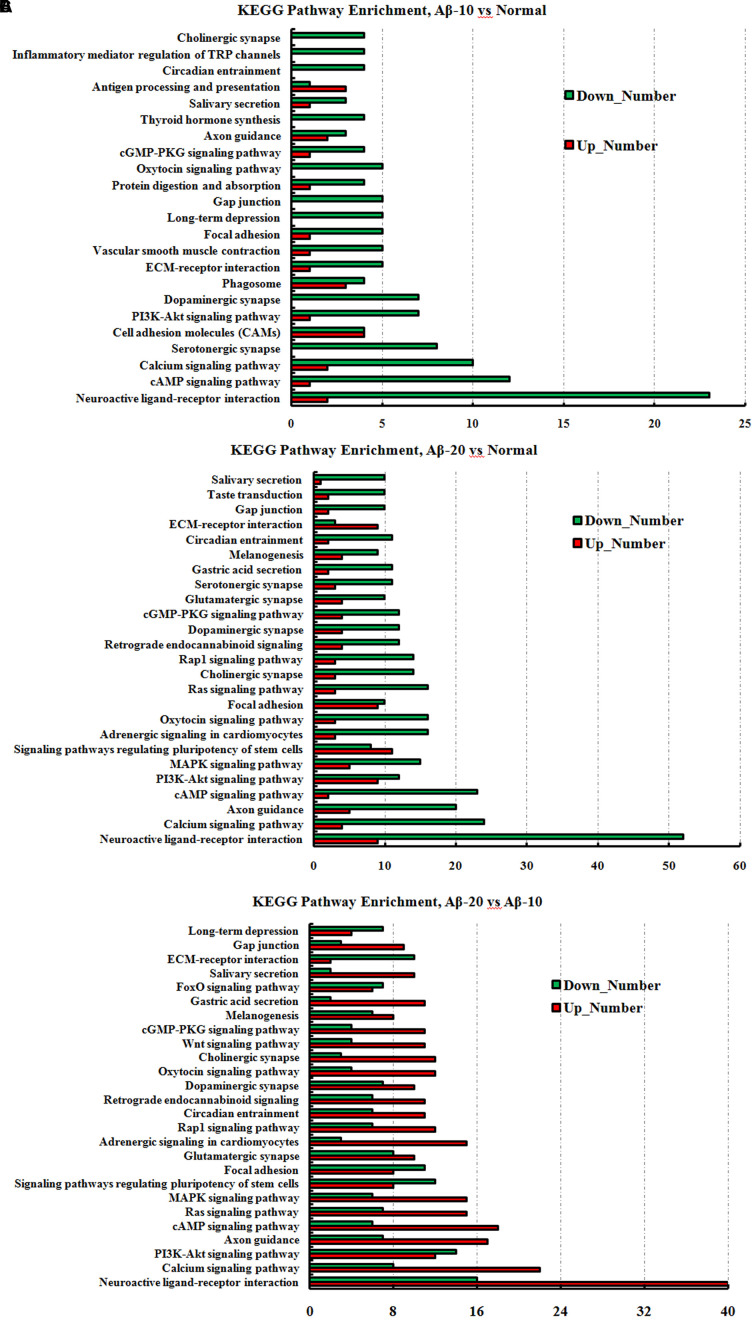
The KEGG pathway enrichment of DEGs in brain transcriptome in the deficit rats induced by Aβ_1-42_. **(A)** The group of Aβ-10 vs. the Normal; **(B)** the group of Aβ-20 vs. the Normal; **(C)** the group of Aβ-20 vs. the Aβ-10.

The GO term enrichment results revealed that the single-multicellular organism process (GO:0044707), nervous system development (GO:0007399), system development (GO:0048731), single-organism developmental process (GO:0044767), multicellular organism development (GO:0007275), developmental process (GO:0032502), generation of neurons (GO:0048699), neurogenesis (GO:0022008), neuron differentiation (GO:0030182), and cell differentiation (GO:0030154) (Supplementary Tables S3-1–S3-3) were influenced after treatment with Aβ_1-42_. In the Aβ_1-42_-20 group, the synapse (GO:0045202), synaptic signaling (GO:0099536), synaptic transmission (GO:0007268), trans-synaptic signaling (GO:0099537), synapse part (GO:0044456), central nervous system development (GO:0007417), and behavior (GO:0007610) were changed more than that in the Aβ_1-42_-10 group, which indicated that the occurrence of AD is dependent on the cumulative amount of Aβ_1-42_. The KEGG pathway results showed that the DEGs were enriched mainly in neuroactive ligand-receptor interaction, cAMP signaling pathway, calcium signaling pathway, serotonergic synapse, PI3K-Akt signaling pathway, dopaminergic synapse, and ECM-receptor interaction (**Figure 13**, additional details were shown in Supplementary Tables S4-1–S4-3), all which indicated that the Aβ_1-42_ levels can significantly influence the brain function.

## Discussion

Increasing evidence suggests that the microbiota-gut-brain axis plays a key role in regulating brain functions, and prebiotics are widely considered to have potential as modulators of brain dysfunctions; however, only limited studies are yet available. Herein, we reported that fructooligosaccharides from *M. officinalis* could markedly modify the behavior, improve oxidative stress and inflammation disorder, regulate the synthesis and secretion of neurotransmitter, ameliorate the swelling of brain tissues, and reduce neuronal apoptosis. We also reported that OMO administration alters the diversity and stability of the microbial community, the expression of the genes of AD intracellular markers such as Tau and Aβ_1-42_. In addition, OMO administration exerted an adequate prebiotic role in regulating the composition and metabolism of gut microbiota in an overdose antibiotics-treated IBD mice model.

Gut microbiota plays a major role in maintaining normal physiological functions in the host. The changes in gut microbiota can lead to changes in brain function, thereby affecting the host behavior (Vuong et al., 2017). Recent studies showed a significant correlation between the changes in gut microbiota and cognitive behavior (Dinan and Cryan, 2017). The modulation of gut microbiota by germ-free animals, probiotics or antibiotics intervention, and fecal microbiota transplantation (FMT) can influence the cognitive behavior of the host (Hu et al., 2016). Our data were in agreement with previous studies, showing that the gut microbiota in two AD-like model rats was altered as compared to the normal rats, as the abundance of *Clostridia* and *Clostridiales* in Aβ_1-42_-induced groups increased significantly (*p* < 0.05), while the groups administered OMO can reverse those changes, especially the probiotic *Lactobacillus* and *Akkermansia* increased distinctly (*p* < 0.05).

The probiotic administration had a marked effect on the cognitive behavior. The prototype probiotic bacterium has been found to up-regulate the hormone oxytocin and systemic immune responses in order to achieve a broad range of health benefits involving wound healing, mental health, metabolism, and myoskeletal maintenance (Servin, 2004; Erdman and Poutahidis, 2016). Studies showed that *Lactobacillus pentosus* var. plantarum C29 from kimchi, a traditional food manufactured by fermenting vegetables (Park et al., 2014), was beneficial to health. It can also protect the memory deficits by inducing the expressions of BDNF and p-CREB in scopolamine-induced memory-deficient mice (Jung et al., 2012), anti-inflammatory amelioration of age-dependent memory impairment in Fischer 344 rats (Jeong et al., 2015), and ameliorate memory impairment and inflammation in D-galactose-induced accelerated aging mouse (Woo et al., 2014). Another study also showed that *L. plantarum* could attenuate anxiety-related behavior and protect against stress-induced dysbiosis in adult zebrafish (Davis et al., 2016). *Lactobacilli* and *Bifidobacteria* exhibited antagonistic activities against microbial pathogens (Servin, 2004). Our data were in agreement with previous studies showing that *Lactobacillus* can ameliorate memory deficiencies (**Figure 4H**); the relative abundance of Aβ_1-42_-induced *Lactobacillus* was reduced starkly, especially in the high-dose group. As a result of OMO administration, the relative abundance of *Lactobacillus* was increased significantly; also, the MWM tests showed that the learning and memory abilities were improved (**Figure 3B**). These results suggested that OMO can promote the abundance of *Lactobacillus* and ameliorate the memory deficiencies.

Fructo-oligosaccharides (FOS) are commonly regarded as a type of prebiotics, favorably stimulating the growth of *Bifidobacteria* and *Lactobacilli*. FOS from *Stevia rebaudiana* roots enhanced the growth of specific strains of both *Bifidobacteria* and *Lactobacilli*, especially, with respect to their fermentation ability (Sanches Lopes et al., 2016). FOS are reserve carbohydrates with important positive health effects and technological applications in the food industry. Another previous study indicated that short-chain fructooligosaccharides could be used optimally in combination with *Bifidobacterium animalis* or *B. longum* strains for the development of synbiotic foods or dietary supplements (Valdés-Varela et al., 2017). Our results showed that FOS from *M. officinalis* also enhances the growth of some probiotics similar to that of *Bifidobacteria* and *Lactobacilli.*

The microbiota can modulate the changes in the gut barrier as well as metabolic and inflammatory responses. Gut barrier function is a key to maintaining a balanced response between the host and its microbiome. This highly complex system involves numerous microbiota-derived factors. *Akkermansia muciniphila* is positively correlated with a lean phenotype, reduced body weight gain, amelioration of metabolic responses, and restoration of gut barrier function is effectuated by the modulation of mucus layer thickness (Bland, 2016; Derrien et al., 2016; Greer et al., 2016; Henning et al., 2017; Van Herreweghen et al., 2017). Our data in D-galactose-induced deficient rats showed that the *Akkermansia* were increased (*p* < 0.05, **Figure 2D**), and the intestinal pathological tissue changes (**Figure 1E**) were improved after administration of OMO, thereby indicating that OMO administration can improve the gut barrier function via targeting the abundance of *Akkermansia*, but need more studies.

Increased gut permeability (leaky gut) and alterations in gut microbiota are now widely accepted as an important link with the etiology, course, and treatment of several neuropsychiatric disorders (Anderson et al., 2016). Gut microbiota-released LPS contributes to chronic inflammation and oxidative stress (Le Sage et al., 2017). Moreover, inflammation was first implicated in AD pathology and development, with the neuropathological findings of activated inflammatory cells (microglia and astrocytes) and inflammatory proteins (for example, cytokines and complement), surrounding the amyloid plaques and the nerve fiber tangles (Alkasir et al., 2017). Our study showed that OMO administration could reduce the levels of LPS in TNBS-induced IBD mice and some pro-inflammatory cytokines in both Aβ_1-42_ induced deficit rats (**Figure 3C**) and TNBS-induced IBD mice (**Figures 5C**, **6**). On the other hand, it can increase the levels of some anti-inflammatory cytokines, which suggested that the administration of OMO (prebiotics) can improve the host inflammatory immune response. The data also showed that OMO administration could enhance the oxidative stress, similar to elevated SOD, MDA, CAT, and inhibiting the MDA production in the D-galactose-induced deficit rats (**Figure 1D**). The gut microbiota is known to play a vital role in those responses (Vanegas et al., 2017), although not fully understood. Taken together, we can summarize that OMO administration influences the host inflammatory immune response and oxidative stress by regulating the gut microbiota.

Preliminary data suggested that FOS increases fecal *Bifidobacteria*, induce immunoregulatory dendritic cell (DC) responses, and reduce the disease activity in patients with Crohn’s disease (Benjamin et al., 2011). Moreover, *Bifidobacteria* are predominant bacterial species in the human gut microbiota and have been considered to exert a beneficial effect on human health by maintaining the equilibrium of the resident organisms. *B. longum* with FOS reduces TNF-α, CRP, serum AST levels, HOMA-IR, serum endotoxin, steatosis, and the non-alcoholic steatohepatitis activity index significantly (Malaguarnera et al., 2012). Human milk contains *B. breve*, *Streptococcus thermophilus*, and short-chain galactooligosaccharides/long-chain fructooligosaccharides with pectin-derived acidic oligosaccharides conferring a protective role against gastrointestinal infections: ameliorating the AD symptoms, modulating the immune response, binding the viral particles, and protecting against rotavirus infection (Rigo-Adrover et al., 2017). The combination of fermented formula with short-chain galactooligosaccharides and long-chain fructooligosaccharides was well-tolerated showing a low overall crying time, low incidence of infantile colic infection, and a stool-softening effect in healthy term infants (Vandenplas et al., 2017). After a broad spectrum antibiotics treatment, the IBD mice model showed that FOS from *M. officinalis* also increases fecal *Bifidobacteria*, ameliorates the symptoms of IBD, and modulates the immune response; the Aβ_1-42_ induced deficient rats showed a similar effect.

The gut microbiota can regulate the activity in the peripheral and central nervous system by various means of communication including vagal nerve and adrenergic nerve activation as well as producing several molecular candidates such as neurotransmitters, neuropeptides, endocrine hormones, and immunomodulators. Host stress hormones, such as noradrenaline, might affect the bacterial activities or signal between bacteria may change the microbial diversity and actions of the gut microbiota. However, these bacteria are capable of synthesizing and releasing several neurotransmitters and neuromodulators or eliciting the synthesis and release of neuropeptides from enteroendocrine cells. Previous studies showed that *Lactobacillus* and *Bifidobacterium* species could produce short-chain fatty acids; *Escherichia*, *Bacillus*, and *Saccharomyces* spp. can produce norepinephrine; spore-forming microbes can produce 5-HT; *Bacillus* can produce dopamine, and *Lactobacillus* can produce acetylcholine (Wall et al., 2014; Potgieter et al., 2015; Yano et al., 2015; Alkasir et al., 2017). In this study, the monoamine neurotransmitter (NE, DA, 5-HT, and 5-HIAA) levels in the brain tissue were reduced in Aβ_1-42_ induced deficient rats, and the OMO administration can reverse this decreasing tendency. Thus, we can conclude that OMO influences some bacteria that affect the synthesis and release of some neurotransmitters and neuromodulators. Similar effects were observed in mice subjected to chronic stress, where the observed behavioral, neurochemical, genetic, and neuroendocrine changes after prebiotic (fructooligosaccharides and galactooligosaccharides) administration could be mediated partially by short chain fatty acids (SCFAs) (Burokas et al., 2017); the increased levels of acetate, propionate, and n-butyrate correlated with behavior and gene expression.

We also observed novel changes in microbiota composition, especially the increase in *Bifidobacterium*, immunological enhancement (**Figures 5**, **6**), and gut barrier impairment (**Figure 5**) in TNBS-induced IBD mice. Previous reports showed that *Bifidobacterium* combined with *L. acidophilus*, *L. casei*, and *L. fermentum* for 12 weeks can affect the cognitive function and some metabolic statuses in AD patients (Akbari et al., 2016), and *B. longum* 1714 reduced the stress and improved the memory in healthy volunteers (Allen et al., 2016). The abundance of *Bacteroides* was also increased with OMO administration in the two AD-like animal models (**Figures 3**, **5**). *Bacteroides* are strict anaerobes critical since the initiation of life (Arboleya et al., 2015), and some strains have been used as probiotics. Previous studies have shown that *Bacteroides fragilis* could reverse the autism-like behavior in mice (Hsiao et al., 2013).

**FIGURE 14:**
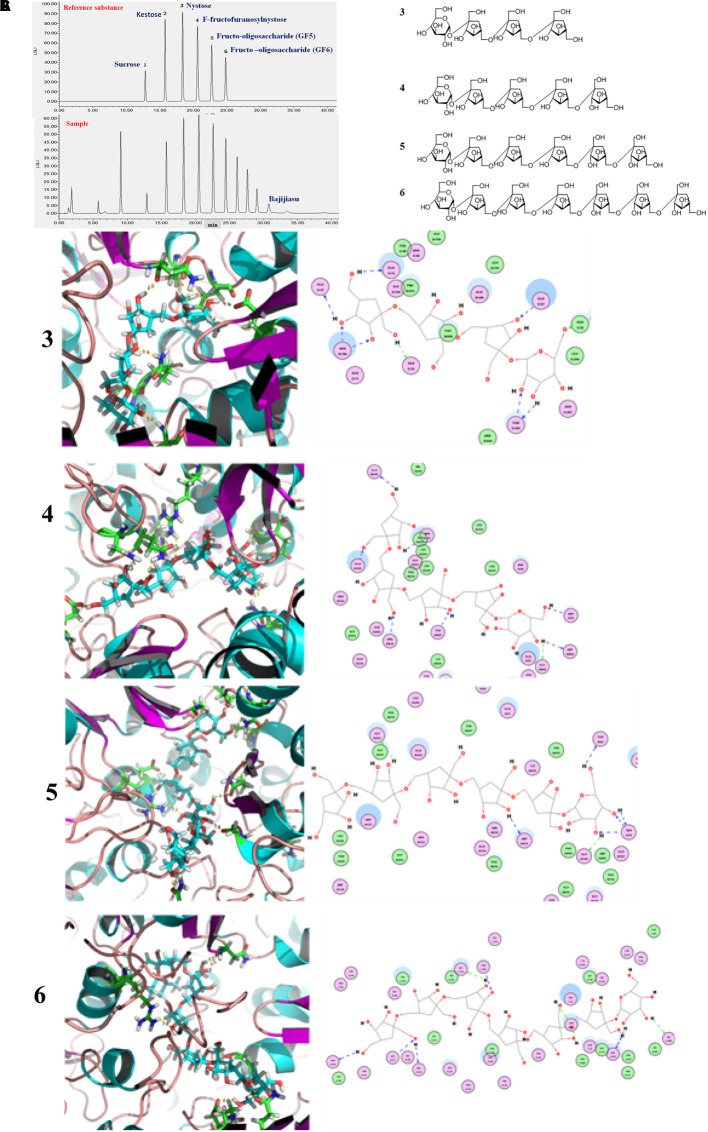
Binding pattern of fructooligosaccharides from *Morinda officinalis*: **(A)** HPLC-ELSD analysis of fructooligosaccharides from *M. officinalis*. **(B)** The chemical structure of nystose (3), *F*-fructofuranose nystose (4), fructooligosaccharide (GF5, 5), fructooligosaccharide (GF6, 6); **(C)** The docked poses were ranked by CDOCKER- ENERGY and the top 10 poses with the co-crystal ligand for SusCD were retained. The data revealed compounds 3, 4, 5, and 6 with CDOCKER-ENERGY of –52.0244180, –75.5881, –101.88, and –110.387, respectively.

The gut microbiota contains highly diverse microbial communities that play a critical role in the metabolic, immunological, and protective functions in health. This phenomenon is influenced by several factors including genetics, host physiology (age of the host, disease, and stress) and environmental factors such as living conditions and use of medications. Increasingly, diet has been recognized as a key environmental factor that mediates the composition and metabolic function of the gut microbiota. Furthermore, the consumption of specific dietary ingredients, such as oil, fibers, and prebiotics, is an avenue that modulates the microbiota. Studies on pigs also suggests that the combination with fructooligosaccharides might represent a valuable symbiotic strategy to increase the probiotic levels of bacteria and survival in the gastrointestinal tracts for feed and food applications (Tanner et al., 2015). The administration of such oligosaccharides is attributable to high interindividual variation of the communities in fecal bacteria from pet cats and dogs (Garcia-Mazcorro et al., 2017). In this study, the fructooligosaccharides were extracted from *M*. *officinalis*, which was widely used in soup, wine, and sweetmeats in South China. Fructo-oligosaccharides are soluble fiber extensively used as prebiotics that is conventionally associated with the stimulation of beneficial bacteria such as *Bifidobacteria* and *Lactobacilli*, among other gut members. However, the mechanisms underlying the fructooligosaccharides stimulation of the beneficial bacteria are yet unknown. A previous study showed that the obtained nutrients process of bacteria require cell membrane protein machines called SusCD complexes (extracellular substrate binding proteins and SusC transporter) (Glenwright et al., 2017), in order to detect the binding capacities between SusCD and nutrients (as starch and other dietary polysaccharides) can evaluate the activity of prebiotics of nutritional ingredients. In this study, the molecular docking analysis was carried out for the evaluation of the binding capacities between SusCD and fructooligosaccharides from *M. officinalis*. The molecular docking study was conducted using the CDOCKER protocol for the four polysaccharides from *M. officinalis* using the Discovery Studio 2.5 (DS2.5) Provisional software. A total of four components (**Figures 14A,B**) from *M. officinalis* were assimilated by literature search, and 1048 poses were generated for all the compounds investigated. The docked poses were ranked by the CDOCKER-ENERGY, and the top 10 poses with the co-crystal ligand for SusCD were retained (**Figure 14**). The data revealed 4 hits namely, compounds 3 (Nystose), 4 (*F*-fructofuranosylnystose), 5 (fructooligosaccharide, GF5), and 6 (fructooligosaccharide, GF6) with CDOCKER-ENERGY -52.0244180, -75.5881, -101.88, and -110.387, respectively (**Figure 14C**). This indicated that the 4 polysaccharides might exert a potent binding activity on SusCD. The interaction between the SusCD protein and the four compounds was further analyzed using the receptor-ligand interaction module in DS. The analysis between SusCD and compound 3 revealed that 9 hydrogen bond interactions appeared in the docked pose. The analysis between SusCD and compound 5 revealed that 9 hydrogen bond interactions appeared in the docked pose. The analysis between SusCD and compound 5 revealed that 7 hydrogen bond interactions appeared in the docked pose. The analysis between SusCD and compound 6 revealed that 9 hydrogen bond interactions appeared in the docked pose. These results suggested that the fructooligosaccharides from *M. officinalis* could be absorbed sufficiently by bacteria with the help of SusCD, serving as optimal nutritional ingredients or prebiotics for bacteria.

**FIGURE 15:**
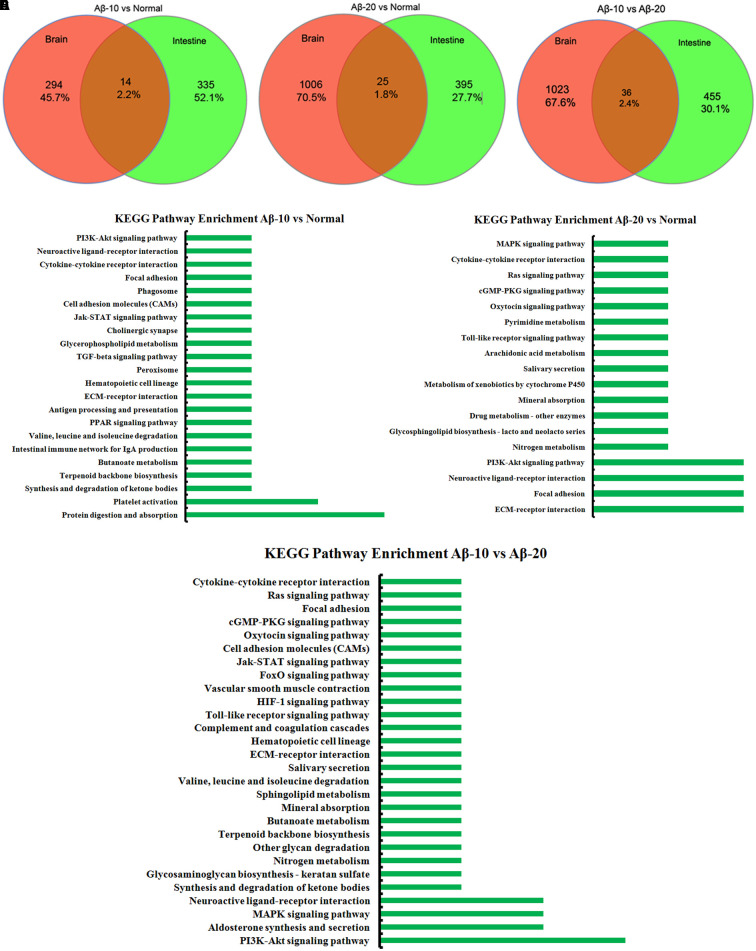
The interactive information analysis of DEGs in brain and intestine in deficient rats by injected Aβ_1-42_. **(A–C)** Wayne chart of DEGs in the brain and intestine transcriptome. **(D–F)** KEGG pathway enrichment of DEGs in the brain and intestine transcriptome.

The changes in behavior and gut microbiota, as a result of different concentrations of Aβ_1-42_, coincided with the alterations in gene expression in critical brain and intestinal regions. The transcriptome analysis of intestine and brain tissues at the 5th week induced by Aβ_1-42_ showed that the expression of some genes changed significantly (**Figures 10**, **12**). Furthermore, to understand the genetic pathways of the gut-brain axis, the significant DEGs both in the brain and intestine were analyzed to identify the gene elements by an interactive information using the Wayne chart (**Figures 15A–C**), which revealed that the interactive genes were increased by the Aβ_1-42_ levels (14 for Aβ-10 vs. normal, while 25 for Aβ-20 vs. normal, *p* < 0.05). The KEGG pathway enrichment analysis showed that the DEGs were primarily enriched in protein digestion and absorption and platelet activation for Aβ-10 induced group (**Figure 15D**). This phenomenon might be attributed to the colonic bacteria that might not be able to run well or are lost, the non-digestible peptides and proteins (collagen) could not be fermented, and some short-chain fatty acids such as butyrate, propionate, and acetate are deficient, which resulted in platelet activation. Thus, the expressions of Col1a1, Col3a1, and Col14a1 mRNA were changed. With the altered mRNA expression of Lamc2, Sstr3, Nts, Spp1, Il6r, Vip, Kcnk3, and Nr4a1, the PI3K-Akt signaling pathway, neuroactive ligand-receptor interaction, focal adhesion, and ECM-receptor interaction were activated in the Aβ-20-induced group (**Figure 15E**). With the increased levels of Aβ_1-42_ levels, the mRNA expressions of Igsf8, Kcnk3, and Mef2c were changed, and the KEGG pathway enrichment analysis showed that the aldosterone synthesis and secretion and MAPK signaling pathway were influenced (**Figure 15F**). Together with the changes in the gut microbiota community diversity shown in **Figure 8**, and the transcriptome analysis data of intestine and brain, we concluded that the Aβ_1-42_ levels in hippocampus interact the gut and microbiota, although further studies are essential for substantiation.

Antibiotics intervention in APPSWE/PS1ΔE9 mouse model suggests that the diversity of the gut microbiota community can regulate the host innate immunity mechanisms that impact Aβ amyloidosis (Minter et al., 2016). The fecal microbiota transplantation implemented from Aβ precursor protein (APP) transgenic mice to non-transgenic wild-type mice showed a drastically increased level of cerebral Aβ levels, thereby indicating a microbial involvement in the development of Aβ pathology, and microbiota contributes to the development of neurodegenerative diseases (Harach et al., 2017; Liu et al., 2017). We also observed that the diversity in the gut microbiota community altered with the levels of Aβ and the induced time (**Figure 8**). Although the complex networks of communication between the gut microbiota and the brain are not yet fully elucidates, it is clear that prebiotics strongly modulates the ecology of the microbiota. However, the role of the microbial composition and the vast quantity, diversity, and functional capabilities of all these gut microorganisms on the brain and behavior are yet to be determined.

## Conclusion

Taken together, these data provide further evidence for a beneficial role of fructooligosaccharides (prebiotics) from *M. officinalis* and the effects on microbiota-brain-gut axis in AD, but need more studies. This study characterized OMO as a promising naturally occurring chemical constituent and suggested microbiota-brain-gut axis as a putative new therapeutic target for the treatment of various neurological diseases by using *M. officinalis* in conventional medicine.

## Ethics Statement

The animal protocols used in this work were approved by the Institutional Animal Care and Use committee of the Center of Laboratory Animals of the Guangdong Institute of Microbiology (GT-IACUC20160426).

## Author Contributions

DC designed the study, carried out the computational analyses and wrote the manuscript. JY and GL collected animal physiological data and fecal samples and extracted ruminal DNA. XY and XT collected animal physiological data and brain samples. DC, TY, and OS collected data regarding the microbial metabolic networks and transcriptome analysis. YX and QW helped to design the study and to develop the metagenomic analysis tools and wrote the manuscript. GZ helped with computational tool development and statistical analyses (PCA). All authors read and approved the final manuscript.

## Conflict of Interest Statement

The authors declare that the research was conducted in the absence of any commercial or financial relationships that could be construed as a potential conflict of interest.

## Footnotes

1
http://deweylab.biostat.wisc.edu/rsem/

2
http://www.bioconductor.org/packages/2.12/bioc/html/edgeR.html

3
https://github.com/tanghaibao/Goatools

4
http://kobas.cbi.pku.edu.cn
