# Corrigendum: Prebiotic effect of fructooligosaccharides from *Morinda officinalis* on Alzheimer's disease in rodent models by targeting the microbiota-gut-brain axis

**DOI:** 10.3389/fnagi.2025.1593725

**Published:** 2025-05-14

**Authors:** Diling Chen, Xin Yang, Jian Yang, Guoxiao Lai, Tianqiao Yong, Xiaocui Tang, Ou Shuai, Gailian Zhou, Yizhen Xie, Qingping Wu

**Affiliations:** ^1^State Key Laboratory of Applied Microbiology Southern China, Guangdong Provincial Key Laboratory of Microbial Culture Collection and Application, Guangdong Open Laboratory of Applied Microbiology, Guangdong Institute of Microbiology, Chinese Academy of Sciences, Guangzhou, China; ^2^Department of Pharmacy, The Fifth Affiliated Hospital of Guangzhou Medical University, Guangzhou, China; ^3^Guangxi University of Chinese Medicine, Nanning, China; ^4^Guangdong Yuewei Edible Fungi Technology Co., Ltd., Guangzhou, China

**Keywords:** fructooligosaccharides, prebiotics, Alzheimer's disease, behavior, microbiota-gut-brain axis

In the published article, there was an error in Figure 3E and Figure 6 as published. The H&E image of the brain of OMO-100 in Figure 3E was used incorrectly, and the IHC images in Figure 6 were misused. The corrected Figure 3 and Figure 6 and their captions appear below.

**Figure 3:**
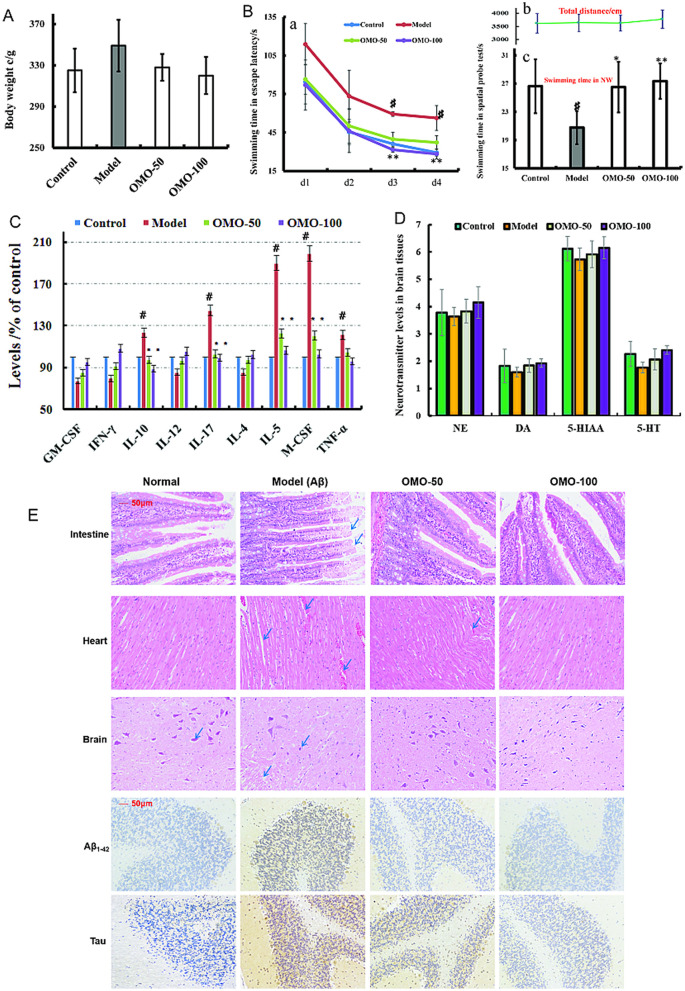
Effect of OMO in Aβ1-42-induced deficient rats. **(A)** Body weight changes during the treatments time. **(B-a)** Escape latency in the MWM. **(B-b)** Swimming distance. **(B-c)** Swimming time in the platform quadrant during the spatial probe test. **(C)** Level of cytokines GM-CSF, TNF-γ, 1L-10, IL-12, 1L-17α, 1L-4, TNF-α, and VGEF-α in the serum. **(D)** Levels of monoamine neurotransmitters (NE, DA, 5-HT, and 5-HIAA) in the brain tissue. **(E)** Histopathological changes in the intestine, heart, and brain, and the expressions of Aβ_1 − 42_ and Tau proteins in brain tissues by immunohistochemistry. The graph Control, control group; Model, model group; OMO-50 mg, low-dose group that received D-galactose (100 mg/kg/d) i.p. and gavage at a dosage of 50 mg/[kg·d] in OMO; OMO-100 mg, high-dose group that received D-galactose (100 mg/kg/d) i.p. and gavage at a dosage of 100 mg/[kg·d] in OMO. Values are represented as mean ± SD (*n* = 6) and expressed as the percentage of the control group, #p < 0.01 vs. control group, ^*^p < 0.05 vs. model group, ^**^p < 0.01 vs. model group.

**Figure 6:**
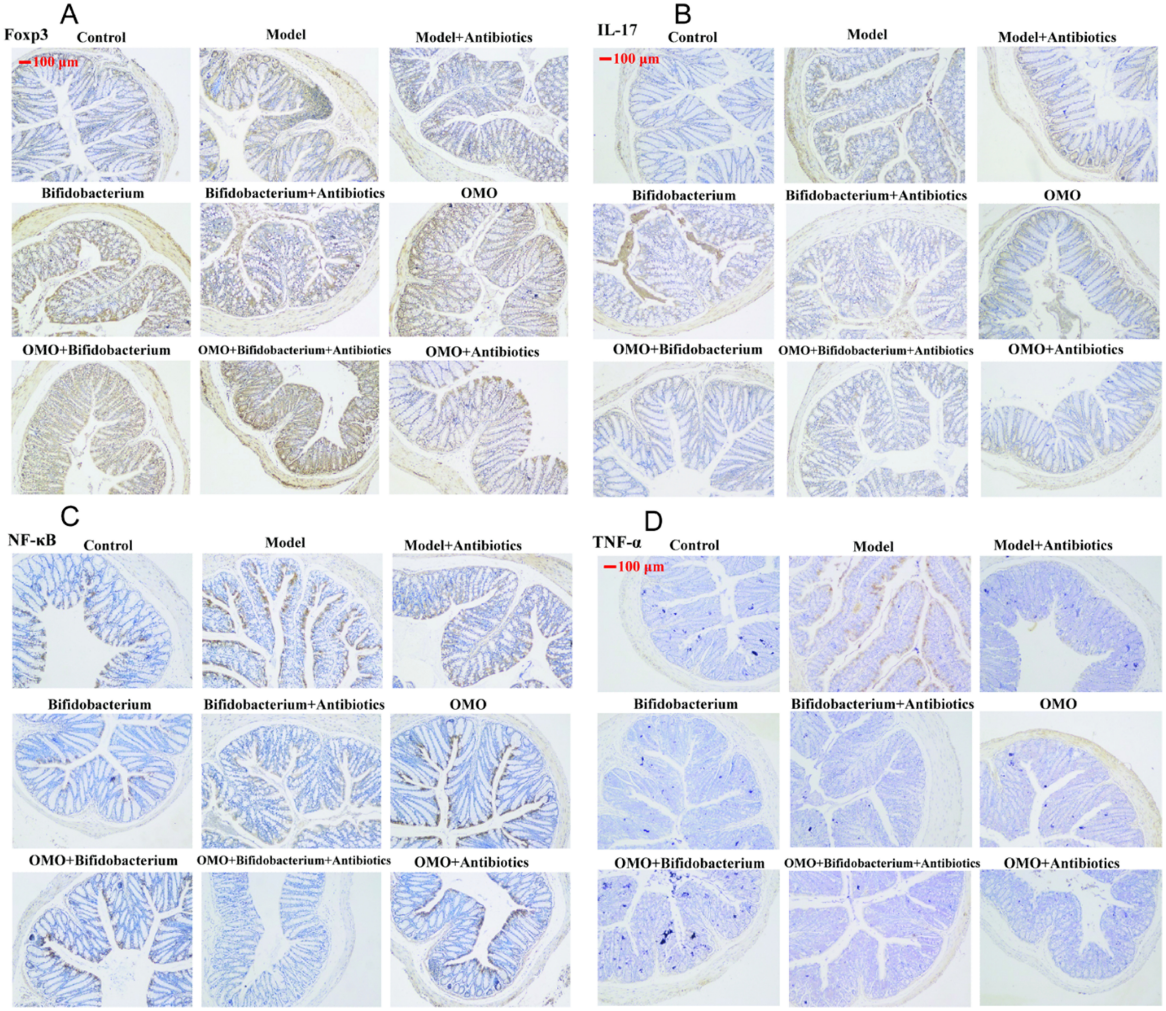
Immunohistochemistry staining of Foxp3 **(A)**, IL-17 **(B)**, NF-κB p65 **(C)**, and TNF-α **(D)** in the colons of different experimental groups in high-dose broad spectrum antibiotics and TNBS-induced IBD mice after treatment with OMO.

The authors apologize for these errors and state that they do not change the scientific conclusions of the article in any way. The original article has been updated.

