# Supplementary material for: Prebiotic Effect of Fructooligosaccharides from Morinda officinalis on Alzheimer’s Disease in Rodent Models by Targeting the Microbiota-Gut-Brain Axis
Source: Front Aging Neurosci. 2017 Dec 8;9:403. doi: 10.3389/fnagi.2017.00403 (PMC5727096; doi:10.3389/fnagi.2017.00403)
Supplement: Supplementary file 1 [file Presentation_1.PDF]

Tab.S1-1. Summary of GO term enrichment results of expression genes in the deficits rat injected different concentration of $A\beta_{1-42}$ (Top 20, Aβ-10 vs normal)

GO_Term	Cluster frequency	Genome frequency of use	Corrected P-value
GO:0006955 immune response	70 out of 328 genes, 21.3%	851 out of 20039 genes, 4.2%	1.41E-26
GO:0005615 extracellular space	80 out of 328 genes, 24.4%	1235 out of 20039 genes, 6.2%	7.36E-25
GO:0002376 immune system process	85 out of 328 genes, 25.9%	1476 out of 20039 genes, 7.4%	4.00E-22
GO:0044421 extracellular region part	127 out of 328 genes, 38.7%	3178 out of 20039 genes, 15.9%	1.15E-21
GO:0005576 extracellular region	134 out of 328 genes, 40.9%	3579 out of 20039 genes, 17.9%	1.50E-20
GO:0031012 extracellular matrix	32 out of 328 genes, 9.8%	338 out of 20039 genes, 1.7%	2.36E-13
GO:0003823 antigen binding	20 out of 328 genes, 6.1%	137 out of 20039 genes, 0.7%	3.00E-11
GO:0048002 antigen processing and presentation of peptide antigen	14 out of 328 genes, 4.3%	53 out of 20039 genes, 0.3%	1.80E-10
GO:0042605 peptide antigen binding	12 out of 328 genes, 3.7%	39 out of 20039 genes, 0.2%	2.43E-10
GO:0042611 MHC protein complex	12 out of 328 genes, 3.7%	43 out of 20039 genes, 0.2%	5.56E-10
GO:0002684 positive regulation of immune system process	38 out of 328 genes, 11.6%	629 out of 20039 genes, 3.1%	7.39E-09
GO:0006952 defense response	45 out of 328 genes, 13.7%	873 out of 20039 genes, 4.4%	1.55E-08
GO:0019882 antigen processing and presentation	15 out of 328 genes, 4.6%	88 out of 20039 genes, 0.4%	2.25E-08
GO:0005578 proteinaceous extracellular matrix	22 out of 328 genes, 6.7%	248 out of 20039 genes, 1.2%	2.74E-08
GO:0002682 regulation of immune system process	48 out of 328 genes, 14.6%	1007 out of 20039 genes, 5.0%	4.55E-08
GO:0006956 complement activation	15 out of 328 genes, 4.6%	93 out of 20039 genes, 0.5%	5.16E-08
GO:0002474 antigen processing and presentation of peptide antigen via MHC class I	11 out of 328 genes, 3.4%	41 out of 20039 genes, 0.2%	6.91E-08
GO:0050778 positive regulation of immune response	27 out of 328 genes, 8.2%	361 out of 20039 genes, 1.8%	1.02E-07
GO:0042612 MHC class I protein complex	9 out of 328 genes, 2.7%	30 out of 20039 genes, 0.1%	1.51E-07
GO:0072376 protein activation cascade	15 out of 328 genes, 4.6%	102 out of 20039 genes, 0.5%	2.01E-07

Tab.S1-2. Summary of GO term enrichment results of expression genes in the deficits rat injected different concentration of Aβ_{1-42} (Top 20, Aβ-20 vs normal)

**GO_Term**	**Cluster frequency**	**Genome frequency of use**	**Corrected P-value**
GO:0002376 immune system process	90 out of 394 genes, 22.8%	1476 out of 20039 genes, 7.4%	2.60E-19
GO:0006955 immune response	62 out of 394 genes, 15.7%	851 out of 20039 genes, 4.2%	5.88E-16
GO:0098552 side of membrane	34 out of 394 genes, 8.6%	410 out of 20039 genes, 2.0%	4.34E-10
GO:0050778 positive regulation of immune response	33 out of 394 genes, 8.4%	361 out of 20039 genes, 1.8%	4.85E-10
GO:0002682 regulation of immune system process	57 out of 394 genes, 14.5%	1007 out of 20039 genes, 5.0%	1.13E-09
GO:0002684 positive regulation of immune system process	43 out of 394 genes, 10.9%	629 out of 20039 genes, 3.1%	2.46E-09
GO:0050776 regulation of immune response	39 out of 394 genes, 9.9%	528 out of 20039 genes, 2.6%	2.75E-09
GO:0009897 external side of plasma membrane	26 out of 394 genes, 6.6%	262 out of 20039 genes, 1.3%	3.76E-09
GO:0006952 defense response	50 out of 394 genes, 12.7%	873 out of 20039 genes, 4.4%	2.32E-08
GO:0043207 response to external biotic stimulus	45 out of 394 genes, 11.4%	730 out of 20039 genes, 3.6%	2.43E-08
GO:0051707 response to other organism	44 out of 394 genes, 11.2%	726 out of 20039 genes, 3.6%	7.31E-08
GO:0009607 response to biotic stimulus	45 out of 394 genes, 11.4%	762 out of 20039 genes, 3.8%	1.01E-07
GO:0046649 lymphocyte activation	26 out of 394 genes, 6.6%	280 out of 20039 genes, 1.4%	1.28E-07
GO:0002768 immune response-regulating cell surface receptor signaling pathway	19 out of 394 genes, 4.8%	151 out of 20039 genes, 0.8%	2.98E-07
GO:0002377 immunoglobulin production	18 out of 394 genes, 4.6%	138 out of 20039 genes, 0.7%	5.01E-07
GO:0003823 antigen binding	17 out of 394 genes, 4.3%	137 out of 20039 genes, 0.7%	5.91E-07
GO:0045321 leukocyte activation	28 out of 394 genes, 7.1%	348 out of 20039 genes, 1.7%	6.65E-07
GO:0002429 immune response-activating cell surface receptor signaling pathway	18 out of 394 genes, 4.6%	142 out of 20039 genes, 0.7%	8.08E-07
GO:0044459 plasma membrane part	74 out of 394 genes, 18.8%	1860 out of 20039 genes, 9.3%	8.25E-07
GO:0002764 immune response-regulating signaling pathway	21 out of 394 genes, 5.3%	200 out of 20039 genes, 1.0%	9.77E-07

Tab.S1-3. Summary of GO term enrichment results of expression genes in the deficits rat injected different concentration of $A\beta_{1-42}$ (Top 20, Aβ-10 vs Aβ-20)

GO_Term	Cluster frequency	Genome frequency of use	Corrected P-value
GO:0002376 immune system process	105 out of 472 genes, 22.2%	1476 out of 20039 genes, 7.4%	7.12E-22
GO:0002682 regulation of immune system process	85 out of 472 genes, 18.0%	1007 out of 20039 genes, 5.0%	7.49E-22
GO:0002684 positive regulation of immune system process	61 out of 472 genes, 12.9%	629 out of 20039 genes, 3.1%	8.81E-18
GO:0050776 regulation of immune response	51 out of 472 genes, 10.8%	528 out of 20039 genes, 2.6%	2.34E-14
GO:0050778 positive regulation of immune response	42 out of 472 genes, 8.9%	361 out of 20039 genes, 1.8%	2.70E-14
GO:0048583 regulation of response to stimulus	135 out of 472 genes, 28.6%	2872 out of 20039 genes, 14.3%	9.00E-13
GO:0046649 lymphocyte activation	35 out of 472 genes, 7.4%	280 out of 20039 genes, 1.4%	1.68E-12
GO:0005576 extracellular region	153 out of 472 genes, 32.4%	3579 out of 20039 genes, 17.9%	2.17E-12
GO:0005615 extracellular space	76 out of 472 genes, 16.1%	1235 out of 20039 genes, 6.2%	2.74E-12
GO:0048584 positive regulation of response to stimulus	88 out of 472 genes, 18.6%	1545 out of 20039 genes, 7.7%	1.26E-11
GO:0045321 leukocyte activation	37 out of 472 genes, 7.8%	348 out of 20039 genes, 1.7%	4.52E-11
GO:0044421 extracellular region part	137 out of 472 genes, 29.0%	3178 out of 20039 genes, 15.9%	5.91E-11
GO:0006955 immune response	60 out of 472 genes, 12.7%	851 out of 20039 genes, 4.2%	6.63E-11
GO:0044459 plasma membrane part	95 out of 472 genes, 20.1%	1860 out of 20039 genes, 9.3%	7.53E-11
GO:0048518 positive regulation of biological process	179 out of 472 genes, 37.9%	4548 out of 20039 genes, 22.7%	8.04E-11
GO:0002253 activation of immune response	29 out of 472 genes, 6.1%	222 out of 20039 genes, 1.1%	1.73E-10
GO:0098552 side of membrane	37 out of 472 genes, 7.8%	410 out of 20039 genes, 2.0%	7.95E-10
GO:0009986 cell surface	49 out of 472 genes, 10.4%	684 out of 20039 genes, 3.4%	1.20E-09
GO:0009897 external side of plasma membrane	29 out of 472 genes, 6.1%	262 out of 20039 genes, 1.3%	1.37E-09
GO:0001775 cell activation	39 out of 472 genes, 8.3%	432 out of 20039 genes, 2.2%	1.72E-09

Tab.S2-1. Summary of KEGG pathway results of expression genes in the deficits rat injected different concentration of Aβ_{1-42} (Top 20, Aβ-10 vs normal)

Pathway	10A_Up_Number	10A_Down_Number	DEG_Number	Total_Number	p-value	FDR
Phagosome	5	12	17	160	6.38E-10	4.12E-08
Antigen processing and presentation	6	9	15	75	6.12E-13	7.90E-11
Cell adhesion molecules (CAMs)	4	10	14	147	8.49E-08	3.65E-06
PI3K-Akt signaling pathway	4	8	12	313	0.004088	0.039416
Endocytosis	3	9	12	261	0.000907	0.014625
Cytokine-cytokine receptor interaction	1	9	10	239	0.004642	0.039647
Natural killer cell mediated cytotoxicity	2	8	10	116	1.48E-05	0.000317
Focal adhesion	2	7	9	191	0.003288	0.037373
Protein digestion and absorption	0	9	9	87	9.03E-06	0.000233
Complement and coagulation cascades	0	9	9	77	3.26E-06	0.000105
Neuroactive ligand-receptor interaction	4	4	8	298	0.098304	0.426018
Rap1 signaling pathway	2	4	6	202	0.099074	0.426018
PPAR signaling pathway	3	3	6	75	0.001153	0.014879
ECM-receptor interaction	1	5	6	75	0.001153	0.014879
Osteoclast differentiation	0	5	5	122	0.043281	0.253782
Bile secretion	3	2	5	68	0.004278	0.039416
Circadian entrainment	4	0	4	90	0.053156	0.274286
TGF-beta signaling pathway	0	4	4	82	0.040025	0.253782
Intestinal immune network for IgA production	2	2	4	44	0.004917	0.039647
Mineral absorption	1	3	4	40	0.003477	0.037373

Tab.S2-2. Summary of KEGG pathway results of expression genes in the deficits rat injected different concentration of Aβ_{1-42} (Top 20, Aβ-20 vs normal)

Pathway	20A_Up_Number	20A_Down_Number	DEG_number	Total_number	p-value	FDR
Cytokine-cytokine receptor interaction	13	2	15	239	5.30E-05	0.001483459
Cell adhesion molecules (CAMs)	11	3	14	147	7.96E-07	3.50E-05
Phagosome	9	3	12	160	5.43E-05	0.001483459
Chemokine signaling pathway	10	2	12	173	0.00011501	0.002249082
B cell receptor signaling pathway	11	0	11	69	6.46E-08	5.68E-06
Metabolism of xenobiotics by cytochrome P450	5	5	10	52	4.00E-08	5.68E-06
Intestinal immune network for IgA production	8	1	9	44	1.10E-07	6.44E-06
Endocytosis	6	3	9	261	0.06029665	0.272107959
Neuroactive ligand-receptor interaction	3	6	9	298	0.1127979	0.374574158
Antigen processing and presentation	5	3	8	75	8.39E-05	0.001846851
Leukocyte transendothelial migration	7	1	8	114	0.001449653	0.019626071
Natural killer cell mediated cytotoxicity	6	2	8	116	0.001620004	0.01964589
PI3K-Akt signaling pathway	5	3	8	313	0.2405271	0.548622913
Drug metabolism - cytochrome P450	2	5	7	53	5.90E-05	0.001483459
Hematopoietic cell lineage	7	0	7	77	0.000624918	0.010998552
Ribosome	0	7	7	151	0.02472103	0.144433378
Focal adhesion	6	1	7	191	0.07080411	0.28690992
Retinol metabolism	3	3	6	60	0.000921531	0.014744501
Complement and coagulation cascades	5	1	6	77	0.003339785	0.032655676
Fc gamma R-mediated phagocytosis	6	0	6	79	0.003794599	0.033392471
NF-kappa B signaling pathway	6	0	6	85	0.005435262	0.039858588

Tab.S2-3. Summary of KEGG pathway results of expression genes in the deficits rat injected different concentration of Aβ_{1-42} (Top 20, Aβ-10 vs Aβ-20)

**Pathway**	**X10A_Up_Number**	**X20A_Up_Number**	**DEG_number**	**Total_number**	**pvalue**	**FDR**
Cytokine-cytokine receptor interaction	2	20	22	239	1.88E-08	3.47E-06
Chemokine signaling pathway	1	17	18	173	5.78E-08	5.32E-06
Cell adhesion molecules (CAMs)	0	14	14	147	4.91E-06	0.000129099
PI3K-Akt signaling pathway	4	9	13	313	0.02230741	0.117273241
Hematopoietic cell lineage	0	12	12	77	1.16E-07	7.12E-06
B cell receptor signaling pathway	0	11	11	69	3.09E-07	1.42E-05
Ras signaling pathway	2	9	11	220	0.009958284	0.07329297
Endocytosis	1	10	11	261	0.03087773	0.141243701
Leukocyte transendothelial migration	0	10	10	114	0.000218297	0.003651515
Phagosome	0	10	10	160	0.002983572	0.032140987
Metabolism of xenobiotics by cytochrome P450	4	5	9	52	1.82E-06	6.69E-05
Retinol metabolism	5	4	9	60	6.25E-06	0.000143719
Fc gamma R-mediated phagocytosis	0	9	9	79	6.01E-05	0.001228569
Osteoclast differentiation	0	9	9	122	0.001550505	0.019181166
Intestinal immune network for IgA production	0	8	8	44	4.66E-06	0.000129099
Complement and coagulation cascades	0	8	8	77	0.000293418	0.004499078
Natural killer cell mediated cytotoxicity	0	8	8	116	0.004213499	0.036918277
Axon guidance	0	8	8	172	0.03736443	0.156251253
Focal adhesion	0	8	8	191	0.06182946	0.199589836
Rap1 signaling pathway	0	8	8	202	0.07971867	0.228519649

Tab. S3-1 Summary of GO term enrichment results of expression genes in brain of the deficits rat injected different concentration of Aβ_{1-42} (Top 20, Aβ-10 vs normal)

GO_Term	Cluster frequency	Genome frequency of use	Corrected p-value
GO:0044707 single-multicellular organism process	129 out of 285 genes, 45.3%	4282 out of 20039 genes, 21.4%	2.09E-16
GO:0048513 animal organ development	82 out of 285 genes, 28.8%	2518 out of 20039 genes, 12.6%	3.54E-10
GO:0007275 multicellular organism development	105 out of 285 genes, 36.8%	3742 out of 20039 genes, 18.7%	5.93E-10
GO:0048856 anatomical structure development	110 out of 285 genes, 38.6%	4116 out of 20039 genes, 20.5%	3.00E-09
GO:0048731 system development	96 out of 285 genes, 33.7%	3385 out of 20039 genes, 16.9%	5.95E-09
GO:0044767 single-organism developmental process	113 out of 285 genes, 39.6%	4342 out of 20039 genes, 21.7%	7.23E-09
GO:0032502 developmental process	113 out of 285 genes, 39.6%	4370 out of 20039 genes, 21.8%	1.11E-08
GO:0048699 generation of neurons	49 out of 285 genes, 17.2%	1201 out of 20039 genes, 6.0%	4.12E-08
GO:0051239 regulation of multicellular organismal process	71 out of 285 genes, 24.9%	2203 out of 20039 genes, 11.0%	4.21E-08
GO:0022008 neurogenesis	51 out of 285 genes, 17.9%	1298 out of 20039 genes, 6.5%	5.90E-08
GO:0007399 nervous system development	62 out of 285 genes, 21.8%	1809 out of 20039 genes, 9.0%	9.29E-08
GO:0030182 neuron differentiation	37 out of 285 genes, 13.0%	763 out of 20039 genes, 3.8%	1.40E-07
GO:0009887 organ morphogenesis	37 out of 285 genes, 13.0%	765 out of 20039 genes, 3.8%	1.50E-07
GO:0007267 cell-cell signaling	26 out of 285 genes, 9.1%	406 out of 20039 genes, 2.0%	3.47E-07
GO:0009888 tissue development	50 out of 285 genes, 17.5%	1326 out of 20039 genes, 6.6%	4.04E-07
GO:0030154 cell differentiation	78 out of 285 genes, 27.4%	2676 out of 20039 genes, 13.4%	4.31E-07
GO:0032501 multicellular organismal process	138 out of 285 genes, 48.4%	6198 out of 20039 genes, 30.9%	7.71E-07
GO:0044057 regulation of system process	25 out of 285 genes, 8.8%	395 out of 20039 genes, 2.0%	1.01E-06
GO:0048869 cellular developmental process	80 out of 285 genes, 28.1%	2866 out of 20039 genes, 14.3%	1.86E-06
GO:0009653 anatomical structure morphogenesis	57 out of 285 genes, 20.0%	1739 out of 20039 genes, 8.7%	3.57E-06
GO:0008015 blood circulation	21 out of 285 genes, 7.4%	302 out of 20039 genes, 1.5%	4.89E-06
GO:0003013 circulatory system process	21 out of 285 genes, 7.4%	307 out of 20039 genes, 1.5%	6.56E-06
GO:0005861 troponin complex	5 out of 285 genes, 1.8%	8 out of 20039 genes, 0.0%	7.83E-06

Tab.S3-2 Summary of GO term enrichment results of expression genes in brain of the deficits rat injected different concentration of Aβ_{1-42} (Top 20, Aβ-20 vs normal)

GO_Term	Cluster frequency	Genome frequency of use	Corrected p-value
GO:0007399 nervous system development	220 out of 972 genes, 22.6%	1809 out of 20039 genes, 9.0%	6.51E-36
GO:0044707 single-multicellular organism process	381 out of 972 genes, 39.2%	4282 out of 20039 genes, 21.4%	5.60E-35
GO:0048731 system development	313 out of 972 genes, 32.2%	3385 out of 20039 genes, 16.9%	9.30E-30
GO:0022008 neurogenesis	168 out of 972 genes, 17.3%	1298 out of 20039 genes, 6.5%	2.42E-29
GO:0048699 generation of neurons	159 out of 972 genes, 16.4%	1201 out of 20039 genes, 6.0%	1.14E-28
GO:0007267 cell-cell signaling	86 out of 972 genes, 8.8%	406 out of 20039 genes, 2.0%	2.30E-28
GO:0097458 neuron part	148 out of 972 genes, 15.2%	1108 out of 20039 genes, 5.5%	8.94E-28
GO:0007275 multicellular organism development	328 out of 972 genes, 33.7%	3742 out of 20039 genes, 18.7%	4.33E-27
GO:0045202 synapse	104 out of 972 genes, 10.7%	625 out of 20039 genes, 3.1%	1.73E-26
GO:0099536 synaptic signaling	64 out of 972 genes, 6.6%	243 out of 20039 genes, 1.2%	3.88E-26
GO:0007268 synaptic transmission	64 out of 972 genes, 6.6%	243 out of 20039 genes, 1.2%	3.88E-26
GO:0099537 trans-synaptic signaling	64 out of 972 genes, 6.6%	243 out of 20039 genes, 1.2%	3.88E-26
GO:0044456 synapse part	91 out of 972 genes, 9.4%	501 out of 20039 genes, 2.5%	8.22E-26
GO:0007417 central nervous system development	118 out of 972 genes, 12.1%	785 out of 20039 genes, 3.9%	3.23E-25
GO:0007610 behavior	91 out of 972 genes, 9.4%	506 out of 20039 genes, 2.5%	1.66E-24
GO:0048856 anatomical structure development	343 out of 972 genes, 35.3%	4116 out of 20039 genes, 20.5%	1.83E-24
GO:0044767 single-organism developmental process	356 out of 972 genes, 36.6%	4342 out of 20039 genes, 21.7%	2.19E-24
GO:0007420 brain development	102 out of 972 genes, 10.5%	631 out of 20039 genes, 3.1%	6.45E-24
GO:0060322 head development	105 out of 972 genes, 10.8%	665 out of 20039 genes, 3.3%	7.52E-24
GO:0032502 developmental process	356 out of 972 genes, 36.6%	4370 out of 20039 genes, 21.8%	7.84E-24
GO:0030154 cell differentiation	252 out of 972 genes, 25.9%	2676 out of 20039 genes, 13.4%	1.50E-23
GO:0030182 neuron differentiation	113 out of 972 genes, 11.6%	763 out of 20039 genes, 3.8%	1.96E-23
GO:0043005 neuron projection	117 out of 972 genes, 12.0%	837 out of 20039 genes, 4.2%	4.18E-23

Tab.S3-3 Summary of GO term enrichment results of expression genes in brain of the deficits rat injected different concentration of Aβ_{1-42} (Top 20, Aβ-10 vs Aβ-10)

**GO_Term**	**Cluster frequency**	**Genome frequency of use**	**Corrected p-value**
GO:0044707 single-multicellular organism process	388 out of 993 genes, 39.1%	4282 out of 20039 genes, 21.4%	2.56E-35
GO:0045202 synapse	114 out of 993 genes, 11.5%	625 out of 20039 genes, 3.1%	3.49E-32
GO:0048731 system development	324 out of 993 genes, 32.6%	3385 out of 20039 genes, 16.9%	3.62E-32
GO:0007275 multicellular organism development	342 out of 993 genes, 34.4%	3742 out of 20039 genes, 18.7%	2.35E-30
GO:0044456 synapse part	98 out of 993 genes, 9.9%	501 out of 20039 genes, 2.5%	6.67E-30
GO:0007399 nervous system development	210 out of 993 genes, 21.1%	1809 out of 20039 genes, 9.0%	1.48E-29
GO:0048856 anatomical structure development	359 out of 993 genes, 36.2%	4116 out of 20039 genes, 20.5%	4.15E-28
GO:0032502 developmental process	372 out of 993 genes, 37.5%	4370 out of 20039 genes, 21.8%	2.74E-27
GO:0044767 single-organism developmental process	370 out of 993 genes, 37.3%	4342 out of 20039 genes, 21.7%	3.58E-27
GO:0097458 neuron part	145 out of 993 genes, 14.6%	1108 out of 20039 genes, 5.5%	3.48E-25
GO:0007267 cell-cell signaling	79 out of 993 genes, 8.0%	406 out of 20039 genes, 2.0%	1.29E-22
GO:0044459 plasma membrane part	195 out of 993 genes, 19.6%	1860 out of 20039 genes, 9.3%	2.60E-22
GO:0048513 animal organ development	242 out of 993 genes, 24.4%	2518 out of 20039 genes, 12.6%	3.25E-22
GO:0022008 neurogenesis	152 out of 993 genes, 15.3%	1298 out of 20039 genes, 6.5%	1.88E-20
GO:0098794 postsynapse	65 out of 993 genes, 6.5%	318 out of 20039 genes, 1.6%	2.50E-20
GO:0030154 cell differentiation	247 out of 993 genes, 24.9%	2676 out of 20039 genes, 13.4%	2.97E-20
GO:0099536 synaptic signaling	57 out of 993 genes, 5.7%	243 out of 20039 genes, 1.2%	8.67E-20
GO:0007268 synaptic transmission	57 out of 993 genes, 5.7%	243 out of 20039 genes, 1.2%	8.67E-20
GO:0099537 trans-synaptic signaling	57 out of 993 genes, 5.7%	243 out of 20039 genes, 1.2%	8.67E-20
GO:0048699 generation of neurons	143 out of 993 genes, 14.4%	1201 out of 20039 genes, 6.0%	1.03E-19
GO:0043005 neuron projection	112 out of 993 genes, 11.3%	837 out of 20039 genes, 4.2%	1.16E-19
GO:0051239 regulation of multicellular organismal process	212 out of 993 genes, 21.3%	2203 out of 20039 genes, 11.0%	7.83E-19
GO:0007417 central nervous system development	106 out of 993 genes, 10.7%	785 out of 20039 genes, 3.9%	7.69E-18

Tab.S4-1. Summary of KEGG pathway results of expression genes in the brain of deficits rat injected different concentration of Aβ_{1-42} (Top 20, Aβ-10 vs normal)

Pathway	Up_Number	Down_Number	DEG_number	Total_number	p-value	FDR
Neuroactive ligand-receptor interaction	2	23	25	298	7.01E-13	7.79E-11
cAMP signaling pathway	1	12	13	188	2.34E-06	0.00013
Calcium signaling pathway	2	10	12	170	4.63E-06	0.000171
Serotonergic synapse	0	8	8	116	0.000214	0.00593
Cell adhesion molecules (CAMs)	4	4	8	147	0.001038	0.019196
PI3K-Akt signaling pathway	1	7	8	313	0.070475	0.258247
Dopaminergic synapse	0	7	7	123	0.00164	0.022756
Phagosome	3	4	7	160	0.007006	0.061477
ECM-receptor interaction	1	5	6	75	0.000604	0.013403
Vascular smooth muscle contraction	1	5	6	122	0.007078	0.061477
Focal adhesion	1	5	6	191	0.050194	0.232148
Long-term depression	0	5	5	59	0.001333	0.021145
Gap junction	0	5	5	85	0.006535	0.061477
Protein digestion and absorption	1	4	5	87	0.0072	0.061477
Oxytocin signaling pathway	0	5	5	152	0.060451	0.252585
cGMP-PKG signaling pathway	1	4	5	159	0.070409	0.258247
Axon guidance	2	3	5	172	0.091141	0.289048
Thyroid hormone synthesis	0	4	4	63	0.011289	0.089505
Salivary secretion	1	3	4	70	0.016126	0.105294
Antigen processing and presentation	3	1	4	75	0.02027	0.124996
Circadian entrainment	0	4	4	90	0.036358	0.183444
Inflammatory mediator regulation of TRP channels	0	4	4	107	0.06144	0.252585
Cholinergic synapse	0	4	4	107	0.06144	0.252585

Tab.S4-2. Summary of KEGG pathway results of expression genes in the brain of deficits rat injected different concentration of Aβ_{1-42} (Top 20, Aβ-20 vs normal)

Pathway	Up_Number	Down_Number	DEG_number	Total_number	p-value	FDR
Neuroactive ligand-receptor interaction	9	52	61	298	4.67E-23	8.50E-21
Calcium signaling pathway	4	24	28	170	4.89E-09	4.45E-07
Axon guidance	5	20	25	172	3.89E-07	2.36E-05
cAMP signaling pathway	2	23	25	188	2.09E-06	9.51E-05
PI3K-Akt signaling pathway	9	12	21	313	0.057779	0.208308
MAPK signaling pathway	5	15	20	250	0.012689	0.06613
Signaling pathways regulating pluripotency of stem cells	11	8	19	136	1.68E-05	0.000382
Adrenergic signaling in cardiomyocytes	3	16	19	140	2.55E-05	0.000515
Oxytocin signaling pathway	3	16	19	152	8.00E-05	0.001324
Focal adhesion	9	10	19	191	0.001449	0.0112
Ras signaling pathway	3	16	19	220	0.006911	0.04492
Cholinergic synapse	3	14	17	107	8.25E-06	0.00025
Rap1 signaling pathway	3	14	17	202	0.013011	0.06613
Retrograde endocannabinoid signaling	4	12	16	101	1.57E-05	0.000382
Dopaminergic synapse	4	12	16	123	0.000179	0.002511
cGMP-PKG signaling pathway	4	12	16	159	0.002953	0.02067
Glutamatergic synapse	4	10	14	110	0.000557	0.005073
Serotonergic synapse	3	11	14	116	0.000951	0.007868
Gastric acid secretion	2	11	13	65	7.29E-06	0.00025
Melanogenesis	4	9	13	90	0.00025	0.002935
Circadian entrainment	2	11	13	90	0.00025	0.002935
ECM-receptor interaction	9	3	12	75	0.000161	0.002439
Gap junction	2	10	12	85	0.00053	0.005073
Taste transduction	2	10	12	87	0.000657	0.005692
Salivary secretion	1	10	11	70	0.000351	0.003759

Tab.S4-3. Summary of KEGG pathway results of expression genes in the brain of deficits rat injected different concentration of Aβ_{1-42} (Top 20, Aβ-20 vs Aβ-10)

Pathway	Up_Number	Down_Number	DEG_number	Total_number	p-value	FDR
Neuroactive ligand-receptor interaction	40	16	56	298	6.85E-19	1.29E-16
Calcium signaling pathway	22	8	30	170	4.70E-10	4.42E-08
PI3K-Akt signaling pathway	12	14	26	313	0.004312	0.031181
Axon guidance	17	7	24	172	2.23E-06	0.000105
cAMP signaling pathway	18	6	24	188	1.06E-05	0.000222
Ras signaling pathway	15	7	22	220	0.000845	0.007946
MAPK signaling pathway	15	6	21	250	0.008541	0.053526
Signaling pathways regulating pluripotency of stem cells	8	12	20	136	6.87E-06	0.000184
Focal adhesion	8	11	19	191	0.001964	0.01678
Glutamatergic synapse	10	8	18	110	4.25E-06	0.00016
Adrenergic signaling in cardiomyocytes	15	3	18	140	0.000119	0.001486
Rap1 signaling pathway	12	6	18	202	0.008011	0.051936
Circadian entrainment	11	6	17	90	1.01E-06	6.31E-05
Retrograde endocannabinoid signaling	11	6	17	101	5.27E-06	0.000165
Dopaminergic synapse	10	7	17	123	7.35E-05	0.001327
Oxytocin signaling pathway	12	4	16	152	0.002442	0.019127
Cholinergic synapse	12	3	15	107	0.000163	0.001917
Wnt signaling pathway	11	4	15	137	0.002242	0.018328
cGMP-PKG signaling pathway	11	4	15	159	0.008975	0.054144
Melanogenesis	8	6	14	90	8.59E-05	0.001327
Gastric acid secretion	11	2	13	65	9.72E-06	0.000222
FoxO signaling pathway	6	7	13	123	0.005766	0.04015
Salivary secretion	10	2	12	70	0.000104	0.001402
ECM-receptor interaction	2	10	12	75	0.000206	0.002279
Gap junction	9	3	12	85	0.000672	0.006647
Long-term depression	4	7	11	59	9.18E-05	0.001327
